# Pollen‐based reconstruction reveals the impact of the onset of agriculture on plant functional trait composition

**DOI:** 10.1111/ele.14063

**Published:** 2022-07-11

**Authors:** Annegreet Veeken, Maria J. Santos, Suzanne McGowan, Althea L. Davies, Franziska Schrodt

**Affiliations:** ^1^ School of Geography University of Nottingham Nottingham UK; ^2^ Department of Geography University of Zürich Zürich Switzerland; ^3^ Aquatic Ecology Department Netherlands Institute of Ecology Wageningen the Netherlands; ^4^ School of Geography and Sustainable Development University of St Andrews St Andrews UK

**Keywords:** climate, early agriculture, Holocene, plant functional traits, pollen data

## Abstract

The onset of agriculture improved the capacity of ecosystems to produce food, but inadvertently altered other vital ecosystem functions. Plant traits play a central role in determining ecosystem properties, therefore we investigated how the onset of agriculture in Europe changed plant trait composition using 78 pollen records. Using a novel Bayesian approach for reconstructing plant trait composition from pollen records, we provide a robust method that can account for trait variability within pollen types. We estimate an overall four‐fold decrease in plant size through agriculture and associated decreases in leaf and seed size. We show an increase in niche space towards the resource‐acquisitive end of the leaf economic spectrum. Decreases in leaf phosphorus might have been caused by nutrient depletion through grazing and burning. Our results show that agriculture, from its start, has likely been gradually impacting biogeochemical cycles through altered vegetation composition.

## Graphical abstract

The onset of agriculture improved the capacity of ecosystems to produce food, but inadvertently altered other ecosystem functions. We evaluate how agriculture and climate affect plant functional composition and reveal selection of common traits across agricultural landscapes.

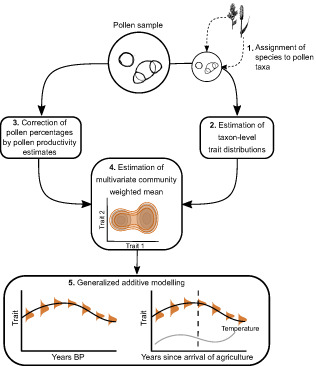

## INTRODUCTION

Recent studies have demonstrated that humans began to alter the world's ecosystems earlier and to a greater extent than previously thought (Ellis et al., 2021; Mottl et al., 2021; Stephens et al., 2019). Agriculture is considered to be the dominant activity through which humans have induced change in ecosystems. In northwestern Europe, forest clearance, burning and land management for agriculture increased openness, heterogeneity and biodiversity of landscapes from 5500 (Central Swiss Alps) and 6000 years ago (British Isles) (Colombaroli et al., 2013; Woodbridge et al., 2020). Agriculture also alters ecological processes. For instance, agriculture may increase erosion and loss of soil organic carbon with such impacts noted as early as 7500 years ago (Hoffmann et al., 2013; Houben, 2008; Notebaert et al., 2009). Plants are central to many ecosystem processes and their variable forms and functions mean that they influence these processes in different ways, including decomposition (Cornwell et al., 2008), carbon storage (Deyn et al., 2008) and erosion control (Zuazo & Pleguezuelo, 2009). Yet little is known about how agriculture changes plant functional composition on timescales of centuries to millennia.

The onset of agriculture may alter plant functional composition in multiple ways. First, the intentional removal of woody species to create agricultural land may have changed plant functional composition. In pre‐agricultural Europe, closed forest most likely dominated fertile upland areas, although open vegetation, maintained by large herbivores and fire, was common on floodplains and infertile soils (Svenning, 2002). Disturbances by agriculture create opportunities for fast‐growing species which disperse rapidly. Especially In the early stages of agriculture, fallowing would have created a heterogeneous agricultural landscape, with areas in use and in various stages of succession (Mazoyer & Roudart, 2007). Second, agriculture can change plant functional composition through the deliberate introduction of species and associated spread of weeds. Crop plants have been selected over millennia for traits that make them more suitable for human consumption and production, such as higher net photosynthesis rates, higher leaf nitrogen content and higher seed production (Roucou et al., 2018).Weed species typically have high growth rates and produce a high number of seeds (Navas, 2012). Finally, agricultural practices can alter plant community functional composition by changing biotic and abiotic factors (Laliberte & Tylianakis, 2012). Even Europe's earliest farmers implemented intensive land management practices, such as livestock manuring and water management (Bogaard et al., 2013).

Whilst land use appears to have strongly controlled northwestern European vegetation composition since the mid‐Holocene, climate was a main driver of vegetation change throughout the Holocene as well (Marquer et al., 2017). 12,000 years ago, mean annual temperature on the Northern Hemisphere was about 2°C lower than the temperature in the 19th century (Kaufman et al., 2020). The increase in temperature at the beginning of the Holocene led to the expansion of mixed deciduous forest, while boreal forest and tundra and steppe vegetation shifted northwards (Roberts, 1998). Maximum forest cover in Europe was reached between 8000 and 6000 BP (i.e. years before 1950) (Roberts et al., 2018). The replacement of tundra and steppe vegetation by forest consequently changed plant functional composition by increasing plant height and other size‐related traits such as leaf size and seed mass (Moles et al., 2009). Further changes in size‐related traits may have occurred through succession within the woodland primarily driven by light competition (Douma et al., 2012). Furthermore, as temperature and water availability are main constraints on the primary productivity of terrestrial ecosystems, rising temperatures in the early Holocene could also have facilitated more competitive species that typically have large leaves and high leaf nutrient concentration (Wright et al., 2017).

The large number of palynological records produced over the last century are invaluable archives of temporal vegetation dynamics. Pollen records have been used to study local or regional history and compiled to reconstruct continental scale vegetation patterns using landscape reconstruction algorithms (see for instance Roberts et al., 2018). Many pollen records are now available in open repositories, most prominently via the Neotoma database (Williams et al., 2018). This allows meta‐analyses which can consider site‐specific factors like bioclimatic zones and timings of different land‐use activities. Classifying pollen according to their functional characteristics or ecological attributes allows comparisons and generalisations across taxa and biomes. In palaeoecology, the classification of pollen types into plant functional types is commonplace, the simplest division being made between arboreal and non‐arboreal pollen (Birks, 2020). More refined plant functional types have been used in vegetation reconstruction by biomisation (Davis et al., 2015) and climate reconstructions using the modern analogue technique (Mauri et al. 2015). However, because plant functional types are relatively coarse categorisations, the range in trait values can be larger within types than between them (Wright et al., 2005). Furthermore, functional types may not capture key plant characteristics that govern relationships between environmental and ecosystem change (Funk et al., 2017; Thomas et al., 2019). In ecology and biogeography, the use of plant traits has become popular in the past two decades, aided by the upsurge in the availability of data on plant traits (Kattge et al., 2020). Functional traits can provide a better understanding of community assembly as well as ecosystem functioning (Hevia et al., 2017). Here, we specifically use the term ‘functional trait’ for traits that influence an organism's fecundity, growth, development or survival and that have been previously defined as representing key dimensions of variation both at the species and the community level (Bruelheide et al., 2018; Díaz et al., 2016; Volaire et al., 2020).

Recently, the trait‐based approach has been applied in several palaeoecological studies to investigate, among other things, the effects of climate and fire on community trait composition (Blaus et al., 2020; Brussel et al., 2018; Carvalho et al., 2019; Reitalu et al., 2015; van der Sande et al., 2019). However, reliable reconstructions of past trait composition are hindered by the general low taxonomic resolution of the palynological record, which can rarely be resolved to the species level (Birks, 2020). As trait data are usually collected at the species level, ascribing trait values to taxa in the pollen record is not straightforward. Trait variation within species, let alone genera or families, can comprise a substantial part of the total trait variation in communities (Siefert et al., 2015). Because of the low taxonomic resolution of pollen data, previous attempts to reconstruct functional diversity from pollen data using mean trait values to represent a whole taxon might have introduced a large amount of uncertainty (Blaus et al., 2020; Carvalho et al., 2019; van der Sande et al., 2019). Here, we offer a new approach to reconstruct functional composition from pollen records. We use Bayesian modelling to consider the full trait distribution within each pollen taxon and account for the consequences of low pollen taxonomic resolution in community‐level trait reconstruction (Bjorkman et al., 2018; Funk et al., 2017).

By reconstructing plant trait composition in multiple pollen records from a variety of different northwestern European locations, we aim to determine trends in plant trait composition within landscapes undergoing the transition to agriculture. We also assess the relative importance of climate versus agriculture in driving these trends. More specifically, we aim to (1) determine whether functional composition exhibits spatial and/or temporal patterns throughout the Holocene in northwestern Europe, (2) examine whether agriculture triggered a change in functional composition and (3) assess the additional role of climate in driving functional composition. We analyse 78 northwestern European pollen records with agricultural histories, spanning the time period from 10,000 BP to the present. Our results provide a first assessment of the changes that the early establishment of agriculture induced in plant trait composition at macroecological scales, which is fundamental to understanding ecological processes of community assembly under human modification.

## METHODS

### Selection of study sites

To identify relevant studies, we conducted a structured search in Web of Science using search terms related to agriculture, the time period and the spatial coverage (full list of search terms is presented in Appendix S1 in Supporting Information). Two hundred and ten publications were retrieved after filtering for relevance. To be included, agriculture had to be identified as one of the drivers of vegetation change in the article, the studies also had to cover more than 500 years and include a dated pollen record. Both arable and pastoral agriculture were considered in the search. We only included studies from which we could retrieve raw pollen data, which left us with 78 sites. The *Neotoma* R package was used to retrieve 74 pollen records from the Neotoma database, in particular its constituent databases the European Pollen Database and the Alpine Pollen Database (Fyfe et al., 2009; Goring et al., 2015; Williams et al., 2018). Pollen records for an additional four sites were supplied by a study author (Davies, 1999). We recorded site characteristics and the start and type of agriculture as defined by the authors of the original studies. An overview of the sites is presented in Appendix S2.

### Preparation of the pollen data

To overcome inconsistencies in pollen nomenclature that exist between laboratories, the pollen nomenclature was harmonised based on the nomenclature of Mottl et al. (2021) and the European Pollen Database (Flantua et al., 2021; Giesecke et al., 2019).

Pollen counts do not precisely represent species abundance because of taxon‐specific differences in pollen productivity and dispersal (Dawson et al., 2016; Seppä, 2013). To correct for this, pollen counts were divided by pollen productivity estimates, which are correction factors obtained from comparison between modern vegetation and pollen surface sediment samples (Bunting et al., 2013). Only taxa with pollen productivity estimates (PPEs) were selected for further analyses (Wieczorek & Herzschuh, 2020, tables 3 and 4) (Appendix S3). On average, this resulted in the exclusion of 2.7% of the total pollen count.

To standardise the radiocarbon dating calibration method across cores, we constructed new chronologies for all records using the *Bchron* R package and the chronological information published in Neotoma (Parnell & Haslett, 2021).

### Attributing species to pollen taxa

**FIGURE 1:**
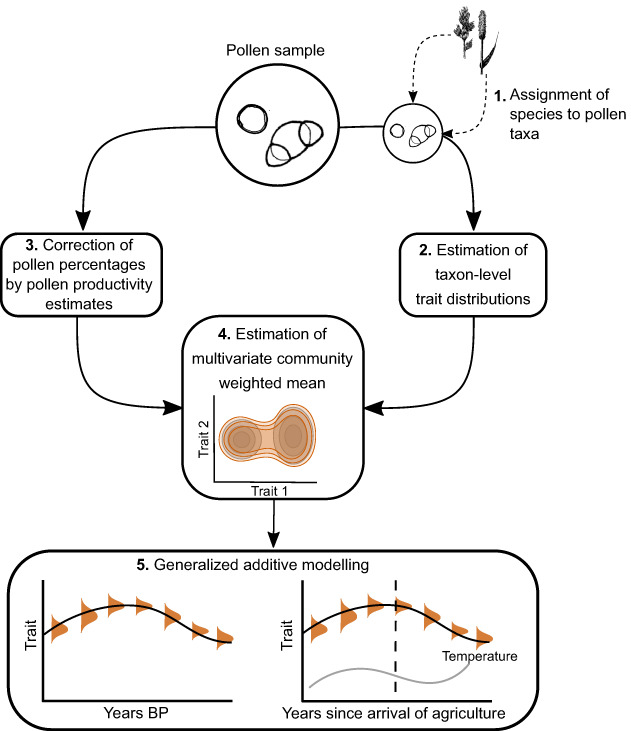
Diagram representing the structure of analysis. 1) Species were assigned to pollen types using the current geographical distribution of the pollen taxon in the GBIF database. 2) Trait distribution at the taxon level was estimated using gap‐filled data trait data from the TRY database. 3) Pollen percentages were corrected by pollen productivity estimates. 4) Trait distribution at the community level was estimated by weighting the taxon estimates by the corrected pollen percentages 5) Community trait values were modelled using generalised additive models.

Trait data are usually collected at the species level, thus to infer trait values at the pollen taxonomic level, we needed to make assumptions about the species belonging to each pollen taxon in our dataset (Figure 1). For this purpose, we made a conversion table for pollen taxon to species from the current distribution of terrestrial species in each pollen taxon in our entire study area. Species distribution data for every pollen taxon in our study area were downloaded from the Global Biodiversity Information Facility database the *rgbif* R package using the pollen taxonomic name and study area's country names as search criteria (Chamberlain et al., 2021; GBIF, 2019). Possible duplicates and incorrectly geo‐referenced observations were cleared from the species distribution data using the *coordinatecleaner* R package, which flags occurrence records to databases of common spatial errors in biological collection data, such as country and province centroids or the location of biodiversity research institutions (Zizka et al., 2019). To improve matching of species names to the trait data, the *taxonstand* R package was used to standardise the species nomenclature according to ‘The Plant List’ (Cayuela et al., 2012; The Plant List, 2013). To filter out (semi)‐aquatic species, we used Ellenberg indicator values for moisture which were downloaded from the TRY database (Ellenberg & Leuschner, 2010; Kattge et al., 2020). Species for which the Ellenberg values were unknown (68%) were retained in the dataset. The resulting conversion table was used for every site in the dataset, and thus included all species found in the geographic range of this study.

### Selection of traits

Ten traits were selected for the analysis: plant height, specific leaf area (SLA), leaf area, leaf nitrogen, leaf phosphorus, leaf carbon, leaf dry matter content (LDMC), seed length, seed mass and seed count. These traits represent key axes of trait variation in plants, as well as main plant strategies that we expect to be affected by the onset of agriculture (Díaz et al., 2016; Grime, 1988; Pierce et al., 2017). Plant height was included because the size of plants and their parts reflect a key dimension of variation in plants (Díaz et al., 2016). The trade‐off between SLA and leaf nutrients on the one hand and LDMC on the other is well established and relates to the resource acquisition strategy of species, referred to as the leaf economic spectrum (Wright et al., 2004). Finally, we included seed traits to represent ruderal species which might benefit from increased disturbance by agriculture. These species are characterised by large leaves with high nutrient content, combined with small and numerous seeds (Grime, 1998; Pierce et al., 2017; Westoby et al., 2002).

We used trait data from the TRY database (Kattge et al., 2020), which is currently the largest global database of plant functional traits. This trait data were previously gap‐filled using Hierarchical Bayesian Probabilistic Matrix Factorisation (HBPMF), an approach specifically developed for plant functional trait imputation (Schrodt et al., 2015). All analyses were done using log‐transformed trait values to approach normally distributed data.

### Calculating community‐weighted mean values

We used a novel Bayesian modelling approach for reconstructing plant trait composition from pollen records. Using the gapfilled trait data of the species that were assigned to the pollen taxa, we first modelled the trait distributions at the pollen taxonomic level. Traits are inherently correlated because organisms have to balance their allocation to survival, growth and reproduction with the availability of resources (Díaz et al., 2016). To allow correlation between traits, we modelled the trait distribution of pollen taxa using a Multivariate Normal distribution for the likelihood, so that

(1) $${\text{trait}}_{i|j}^{\text{observed}}∽{\text{Normal}}_{k}\left(\mu_{j},\sum_{j}\right)$$

where *i* represents a trait observation from pollen taxon *j* from the gap‐filled trait data, and *k* the number of dimensions of the multivariate distribution, corresponding to ten traits in this analysis. ***μ***_***j***_ is a vector of mean trait values of length *k*. ***∑***_***j***_ is a covariance matrix of *k* by *k*. Vague priors were used for the taxon mean and standard deviation estimation, so the posterior is strongly informed by the data. The trait data were Z‐score standardised before modelling.

We then modelled the trait distribution of a community (community weighted mean, CWM) by weighting the taxon trait distributions of each pollen taxon by the corrected pollen percentages at site *s* and moment in time *y*, so that

(2) $${\mathrm{CWM}}_{s,t}∽\sum^{n}\pi_{j}{\text{Normal}}_{k}\;\left(\mu_{j},\sum_{j}\right)\;$$

where *n* is the total number of taxa and *π*_j_ the abundance of pollen taxon at a given site *s* and moment in time *t*. We carried through the mean (*CWM* ^*mean*^ *)* and the standard deviation *(CWM* ^*sd*^ *)* of the modelled distribution in the subsequent analyses to account for inherent uncertainties in estimations of the mean weighted trait value.

All Bayesian models were run in JAGS (v4.3.0), a program for Bayesian analysis using Markov Chain Monte Carlo simulation (MCMC). The *runjags* R package provided an interface for running JAGS in R (Denwood, 2016). The performance of the MCMC random walk, that is the chain, to sufficiently represent the posterior was reviewed by checking effective sample size and chain convergence using the *coda* R package (Plummer, 2004; Plummer et al., 2006). The JAGS code for all models can be found in Appendix S4. We also performed the analysis using a univariate Beta for LDMC and log‐Normal likelihood distribution for the other traits (Appendix S5).

### Principal component analysis

To explore variation in the community trait values of all 10 traits, a principal component analysis (PCA) was performed on the mean estimates of CWM *(CWM* ^*mean*^ *)* using the *prcomp* function in R. To investigate spatial and temporal differences in multivariate trait composition, we plotted the sample scores of the first and second principal component for six clusters that were based on the proximity of sites to each other. We ran generalised additive models (GAMs) with a smooth for time and random effect of site for the first two principal components and the six clusters using the *mgcv* package. To test the sensitivity of the analysis to the inclusion of plant height, as a trait that is obviously affected by agriculture, we recalculated *CWM* ^*mean*^ without plant height, and performed the PCA on these values (Appendix S6).

### Trait composition change over time

To examine trends in individual trait values over time, we used Bayesian GAMs. In this analysis, both the estimates of *CWM* ^*mean*^ and *CWM* ^*sd*^ were used. The JAGS code for the GAM was generated using the *jagam* function of the *mgcv* R package and adjusted to include the estimates of *CWM* ^*sd*^ and a random effect of site (*α*_*s*_) (Wood, 2016, 2017).
(3)

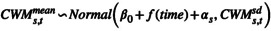

where *f(time)* is the smoothing function for time before present. To show the dispersion of the reconstructed trait values and give some insight in to species compositional changes, we plotted the reconstructed community trait values as points together with the modelled trends and coloured them by the proportion of trees. JAGS code for the GAMs can be found in Appendix S4. Model fit was evaluated by simulating datasets from the posterior as done in Simpson (2018) and Wood (2016). Robustness of the trends to the choice of sites was tested by running the GAMs, while leaving one site out at the time. Effect of uncertainty in the radiocarbon dating was evaluated by running the GAMs using 50 randomly sample age distributions from the posterior of the *Bchron* age models. These simulations can be found in Appendix S7.

### Effects of agriculture and climate on trait composition

We assessed relationships between the arrival of agriculture and changes in CWM by fitting another GAM with a smoothing function for the time since the arrival of agriculture *(f[agriculture])*. Where possible, the start of agriculture at each site was retrieved from the associated publication and was thus based on the original author's expert knowledge of the pollen record, other palaeoecological records and archaeological findings, depending on the study. When this information was not clearly defined in the publication, the EUROEVOL and the ArchaeoGLOBE databases were used to distinguish the before and after agriculture time period in the records. The EUROEVOL dataset consists of radiocarbon‐dated archaeological findings from Neolithic Europe and the ArchaeoGLOBE holds consensus data on the extent of agriculture in that region (Manning et al., 2016; Stephens et al., 2019). Eighteen sites were removed from the dataset for this analysis as they already had agriculture present before the start of the pollen record. To account for climatic change through time, we included a smoothing function for temperature in the model *(f[temperature])*. Mean annual temperature simulations were obtained from CHELSA‐TraCE21k(Karger et al., 2020) (Appendix S8). CHELSA‐TraCE21k is a downscaling algorithm of the TraCE21k palaeoclimate simulations that can generate global temperature estimates at a temporal resolution of 100 years and 30 arcsec spatial resolution for the last 21,000 years. Site was included as a random effect (*α*_*s*_).
(4)

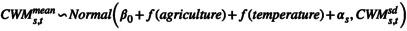

To show the dispersion of the reconstructed trait values and give some insight in to species compositional changes, we plotted the reconstructed community trait values as points together with the modelled trends and coloured them by the proportion of crop species. R version 4.0.5 was used throughout this study (R Core Team, 2021).

## RESULTS

### Site characteristics and pollen taxa

The start of agriculture at the sites ranged between 7000 BP and 750 BP. Arable farming was identified in the majority of sites (69/78). Tree cultivation was performed in 15 sites, mainly in the south of the study area. In four of the 78 sites, primarily in upland areas, pastoralism was the sole type of farming identified. Other important drivers of vegetation change as identified in the original publications were climate (40/78), fire (anthropogenic and natural; 34/78) and woodland clearance for iron smelting (14/78).

Forty‐seven pollen taxa were included in the analysis and a pollen‐type‐species conversion table was made for all of them. In total, the trait data for 2357 species were included in the analysis. An overview of the number of species included in a pollen taxon and the number of trait observations can be found in Appendix S3.

### Spatial and temporal differences in multiple traits

**FIGURE 2:**
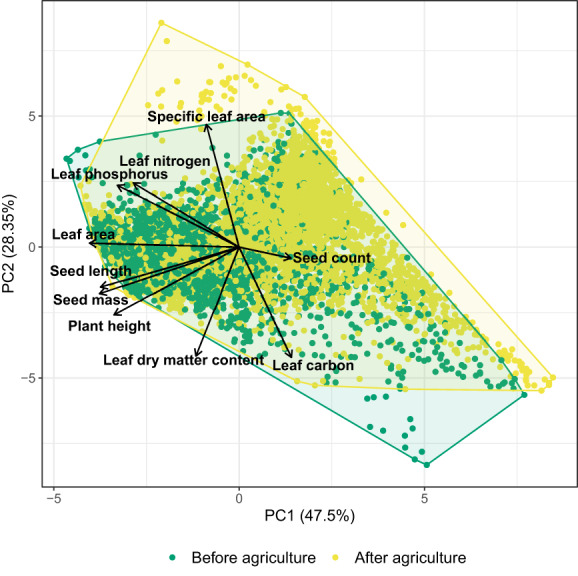
Principal component analysis of community level trait values. Every coloured point represents the reconstructed community trait value. The colour indicates whether the observation is from before or after agriculture. Table 1 Presents the loadings of the traits on the principal components.

The first two components of the PCA account for 75.8% of the total variation *CWM* ^*mean*^ (Figure 2, Table 1). The first principal component (47.5%) mainly represents the variation in plant size and their organs, with the strongest contribution of leaf area, seed size and plant height. The second principal component (28.3%) expresses variation in traits of the leaf economic spectrum, where communities of high specific leaf area and high leaf nutrients are found on one end of the spectrum and communities of high leaf carbon and LDMC at the opposite end. Seed count is the strongest contributor to the third component (10%) (Appendix S6). The strongest positive correlation was found between size‐related traits, such as seed mass and seed length (*r* = 0.96, 95% credibility interval; [0.957, 0.961]) and seed mass and leaf area (*r* = 0.88 [0.87, 0.89]). Correlation was also high between leaf traits, such as leaf phosphorus and leaf area (*r* = 0.75 [0.74, 0.76]). Leaf carbon and specific leaf area were negatively correlated (*r* = −0.71 [−0.72, −0.7]) (Appendix S9).

**TABLE 1:** Loadings of traits on the principal components

Trait	PC1	PC2	PC3
Leaf area	−0.449	0.017	0.034
Seed mass	−0.419	−0.198	0.037
Seed length	−0.416	−0.17	0.001
Plant height	−0.375	−0.288	−0.103
Leaf phosphorus	−0.365	0.262	0.173
Leaf nitrogen	−0.317	0.272	0.064
Leaf dry matter content	−0.13	−0.463	0.311
Specific leaf area	−0.098	0.52	0.107
Seed count	0.155	−0.047	0.914
Leaf carbon	0.157	−0.468	−0.097
Proportion of variance explained (%)	47.5	28.3	10

**FIGURE 3:**
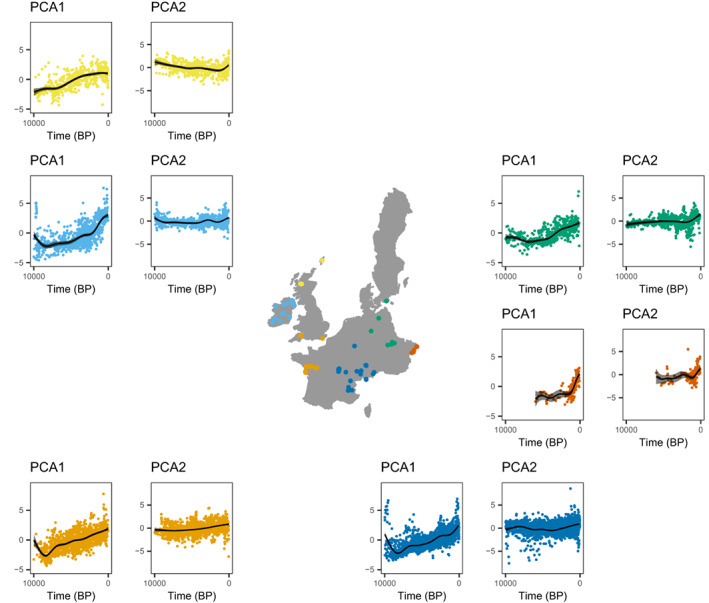
Trends in the first and second principal component over time for six clusters of proximate sites (see Table 1 and Figure 2). The first principal component comprises the variation in plant, leaf and seed size, while the second principal component expresses variation in traits of the leaf economic spectrum.

The lowest scores on the first principal component are found in the early Holocene (10000–6000 BP). Trends in the first principal component are similar and increasing across the study area, indicating a general decrease in community whole plant size. The trend in the second principal component changes from about 4000 BP in the most Southern sites and 2000 BP in the rest of the study area, meaning communities become higher in SLA and lower in leaf carbon (Figure 3).

### Changes in individual traits over time

**FIGURE 4:**
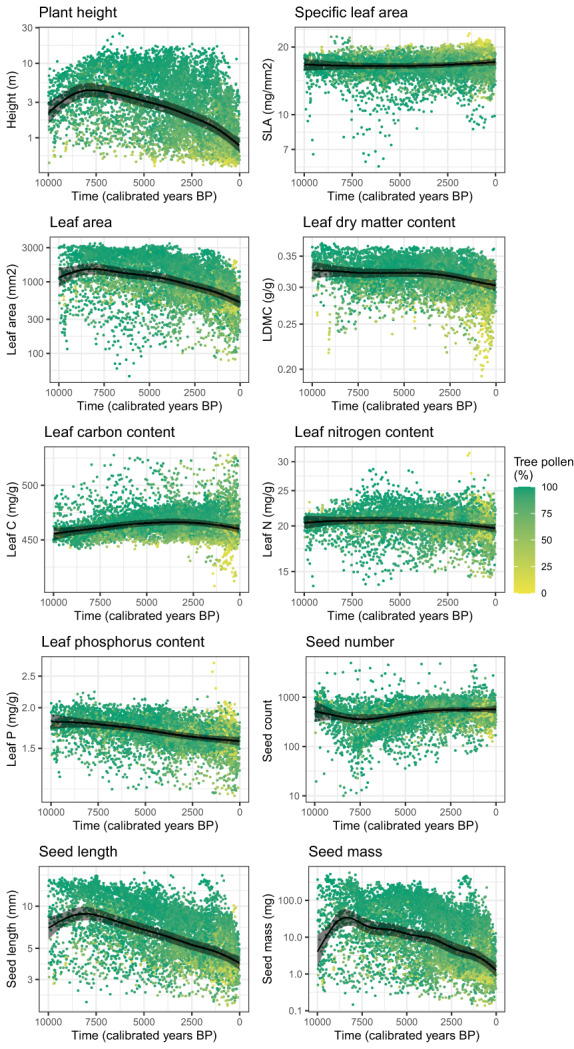
Smooth lines represent the generalised additive model (GAM) of the relationship of CWM of 10 traits with time (calibrated years before present), for all 78 sites. 95% credibility interval is indicated by the shaded area. The reconstructed trait values are plotted and coloured by the corrected proportion of tree pollen at that time point.

Community plant height, leaf area, seed size increased in the first half of the Holocene and decreased from about 7500 BP (Figure 4). Community plant height was on average highest at 7700 BP with 4.3 m [3.5, 5.3], and lowest at the present with 0.76 m [0.6, 1.0]. Leaf phosphorus and LDMC decrease significantly over time. LDMC decreases from 0.33 g/g [0.31, 0.34] to 0.30 g/g [0.29, 0.31]. SLA, leaf carbon, leaf nitrogen and seed number remain stable over time.

### Effects of agriculture and climate on trait composition

**FIGURE 5:**
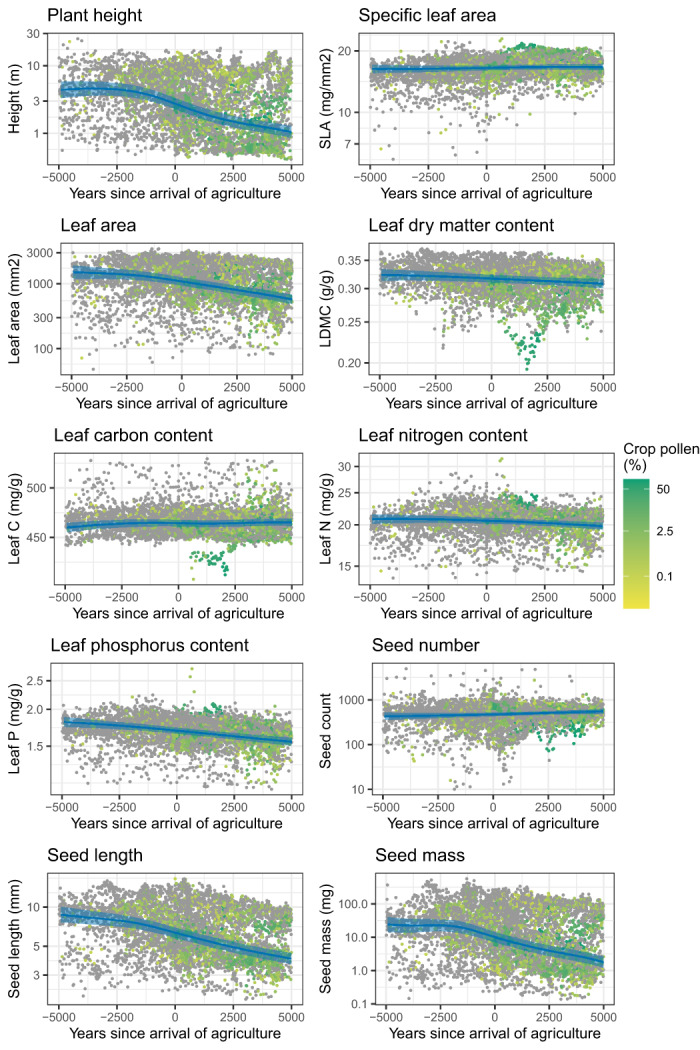
Partial component plots of the relationship between the 10 traits and years since arrival of agriculture. Eighteen sites were removed in this analysis, because agriculture was already present before the start of the records. 95% credibility interval is indicated by the shaded area. The reconstructed trait values are plotted and coloured by the corrected proportion of crop pollen (log transformed) at that time point.

**FIGURE 6:**
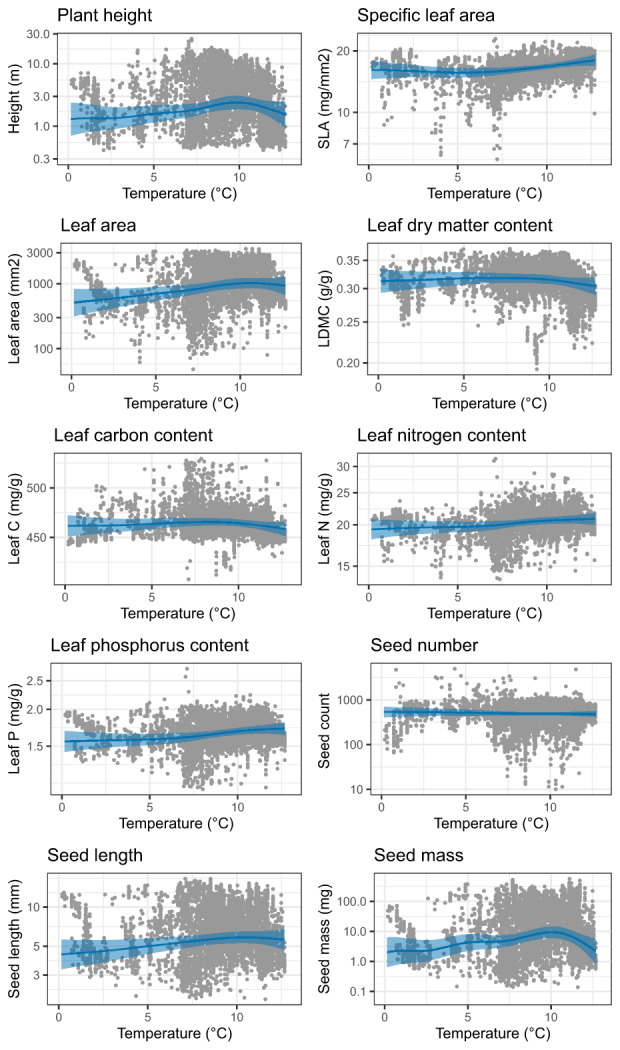
Partial component plots of the relationship between the 10 traits and temperature. Eighteen sites were removed in this analysis, because agriculture was already present before the start of the records. 95% credibility interval is indicated by the shaded area.

Plant height and seed size decrease around the onset of agriculture and onwards (Figure 5), whereas climate had little effect on these traits in the time period investigated in this study (Figure 6). Seed mass changes from 23.8 mg [14.0, 40.4] 5000 years before the arrival of agriculture to 9.5 mg [6.7, 13.4] at the start of agriculture, and to 1.8 mg [1.2, 2.6] 5000 years after the arrival of agriculture. Leaf traits were influenced by climate, with increases in SLA, leaf area and leaf nutrients corresponding with increasing temperature. Leaf area and leaf phosphorus decrease with agriculture, leaf phosphorus from 1.69 mg/g [1.65, 1.73] at the start of agriculture to 1.55 mg/g [1.51, 1.60] at the present. No relationship between the arrival of agriculture and SLA, leaf carbon, leaf nitrogen and LDMC could be detected.

## DISCUSSION

By combining functional trait and pollen data, we have identified changes in functional composition of northwestern European sites after the onset of agriculture. These results are especially important for the understanding human impacts on carbon and nitrogen cycles. We find a general decrease in whole plant size alongside a shift in trait space related to the leaf economic spectrum after the onset of agriculture. The changes in the leaf economic spectrum emerge from a gain of communities with a resource‐acquisitive strategy and a loss of communities with a resource‐conservative strategy (Figure 2). Both agriculture and climate play a role in explaining the changes in plant community trait composition, but the contribution of these factors varies with the different traits (Figures 4, 5 and 6). Plant height and seed size appear to be especially influenced by agriculture and little by climate, whereas leaf traits appear to be influenced by both agriculture and climate.

### Decrease in whole plant size is triggered by the onset of agriculture

The trend in the first principal component demonstrates a common decrease in the size of whole plants, seeds and leaves across the study area (Table 1, Figures 2 and 3). This decrease is likely solely attributable to the onset of agriculture as we found little effect of climate on seed size or plant height (Figure 6). A decrease in plant height is the most intuitive change due to the opening of forest for agriculture, shown in many previous studies (Rey et al., 2019; Roberts et al., 2018). Here, we find an overall four‐fold reduction in plant height in our study area since the start of agriculture (Figure 5). Plant height is the most important predictor of vegetation carbon storage (Conti & Díaz, 2013), thus quantification of the effect of the onset of agriculture on plant height is central to the understanding of human impact on the carbon cycle. Although decreasing plant height with agriculture is not terribly surprising, the fact that we can identify and quantify this trend demonstrates that pollen‐based functional reconstructions can provide reliable insights, even though some pollen types vary widely in plant height (Appendix S10).

We hypothesised that opening up of ecosystems for new agricultural land by fire or deforestation, provides an advantage to species that are fast growing and rapidly dispersed. These species are characterised by low seed size and high seed count to facilitate effective dispersal (Pierce et al., 2017). Although our results show a reduction in seed size with the arrival of agriculture, seed count remains stable over time (Figure 5). We thus find only limited support for this hypothesis. The decrease in seed mass and length with the arrival of agriculture is most likely also due to removal of forests, as these traits are highly correlated (Díaz et al., 2016).

### Communities became more resource‐acquisitive

We show a trend towards the acquisitive end of the leaf economic spectrum, especially in the last 2000 years (Figures 2 and 3). This is likely both driven by agriculture and climate (Figures 5 and 6). With increasing temperature at the beginning of the Holocene, limits on primary productivity were released, giving a competitive advantage to species with traits that support rapid growth and efficient use of available resources (Wright et al., 2017).

We expected that due to the onset of agriculture, communities would become more resource‐acquisitive because of the deliberate manipulation of soil fertility and the characteristics of crop and early successional species (Roucou et al., 2018). The gain in resource‐acquisitive species could have consequences for ecosystem functioning (Lavorel & Grigulis, 2012; Reich, 2014). Acquisitive communities have highly digestible litter and a high availability of nutrients leading to rapid decomposition rates and low litter accumulation (Cornwell et al., 2008). Although some communities after agriculture occupy trait space outside the range found before agriculture, there is a significant overall decrease in leaf phosphorus and leaf area (Figure 5). We think that this seemingly contradictory result is due to differences in the trajectories of agriculture among locations. Crop species as well as other agricultural indicator species (e.g. *Rumex*, *Plantago*) are high in leaf phosphorus content (Appendix S10). However, the depletion of nutrients by grazing, burning and removal of trees favoured the establishment of heathland, especially in Scotland and Ireland, which is low in leaf phosphorus and leaf area (Hjelle et al., 2010; Trondman et al., 2015; Webb, 1998).

### Uncertainties and future directions

Our Bayesian modelling approach for functional composition reconstruction allowed us to account for the uncertainty introduced by the low taxonomic resolution of the pollen records. The method can furthermore be expanded to other traits and can be used for the quantification of functional diversity, which is likely to be an important contributor to ecosystem functioning alongside the dominance of trait values (Garnier et al., 2015). Palaeoecological studies provide a rich array of proxies on past ecosystem functioning, including erosion control and nutrient retention (Bennike et al., 2021; Jeffers et al., 2015). Combining these proxies and functional composition reconstructions could open a new avenue for research into the relationships between plant composition and ecosystem functioning, and the legacies of human presence. While we were able to incorporate the uncertainty caused by the low taxonomic resolution of pollen data, a limitation of this study is that we have not been able to evaluate the effect of trait dispersion within pollen taxa on the community‐level trait reconstruction. Further work will be needed to examine this effect. Better ways of making conversion tables to assign species to pollen taxa could reduce the amount of uncertainty in the reconstructions. For this more information on past species distributions is necessary, for instance from macrofossil records (Birks & Birks, 2000), ancient DNA from lake sediments (Parducci et al., 2017) and species distribution modelling (Svenning et al., 2011). It is also important to reflect that this landscape scale analysis may have masked finer‐scale trends and that we have not been able to study the effect of differences in agricultural method and intensity between sites. Lack of independent data regarding the variable agricultural trajectories at sites made us unable to study these factors. Furthermore, here we included mean annual temperature as the only climate proxy, while other climatic factors as well as soil differences could also be important in explaining plant trait composition (Joswig et al., 2022). However, the approach we developed for reconstruction of plant functional composition can easily be applied in more nuanced analyses. These kinds of analyses will be significantly aided when more pollen records and other palaeoecological proxies are published in public databases.

The palaeoecological record provides a unique long‐term perspective into the legacies of human impact and can, by applying concepts from ecology and biogeography, provide us with valuable insights on the maintenance of ecosystem functioning in a domesticated world. This analysis is the first empirical demonstration of common changes in the plant functional trait composition of landscapes in the transition to agriculture. Our results show that early agricultural might had significant impacts on biogeochemical cycles through modifying vegetation composition, and we show that these impacts can be demonstrated early on.

## AUTHORSHIP

AV designed the study supervised by MJS, SM and FS. AV conducted the analysis with help of FS. AD provided data. AV wrote the manuscript and all authors contributed substantially.

### PEER REVIEW

The peer review history for this article is available at https://publons.com/publon/10.1111/ele.14063

## Supporting information


Data S1

## Data Availability

R‐scripts and the datasets necessary to run the analysis are available in OSF repository (doi: 10.17605/OSF.IO/JK5BF).

## References

[ele14063-bib-0001] Ammann, B. , Oeschger, H. , Andree, A. , Moedl, M. , Rieden, T. , Siegenthaler, U. et al. (1985) Lobsigensee—late‐glacial and Holocene environments of a lake on the central swiss plateau. Introduction and palynology: vegetational history and core correlation at Lobsigensee (swiss plateau). Dissertationes Botanicae, 87, 127–135.

[ele14063-bib-0002] Argant, J. & Argant, A. (2000) Mise en évidence de l'occupation ancienne d'un site d'altitude: analyse pollinique du lac du Lauzon (Drôme). Les Paléoalpins. Hommage à Pierre Bintz, 31, 61–71.

[ele14063-bib-0003] Bennett, K. , Boreham, S. , Sharp, M. & Switsur, V. (1992) Holocene history of environment, vegetation and human settlement on Catta ness, Lunnasting, Shetland. Journal of Ecology, 80, 241–273.

[ele14063-bib-0004] Bennike, O. , Odgaard, B.V. , Moorhouse, H. , McGowan, S. , Siggaard‐Andersen, M.‐L. , Turner, B. et al. (2021) Early historical forest clearance caused major degradation of water quality at Lake Væng, Denmark. Anthropocene, 35, 100302.

[ele14063-bib-0005] Berglund, B.E. , Gaillard‐Lemdahl, M.J. & Göransson, H. (1991) The Bjäresjö area. The cultural landscape during 6000 years in southern Sweden ‐ the Ystad project. 41. Ecological Bulletins, 41, 167–174.

[ele14063-bib-0006] Birks, H.H. & Birks, H.J.B. (2000) Future uses of pollen analysis must include plant macrofossils. Journal of Biogeography, 27, 31–35.

[ele14063-bib-0007] Birks, J.H.B. (2020) Reflections on the use of ecological attributes and traits in quaternary botany. Frontiers in Ecology and Evolution, 8, 166.

[ele14063-bib-0008] Björck, S. & Möller, P. (1987) Late Weichselian environmental history in southeastern Sweden during the deglaciation of the Scandinavian ice sheet. Quaternary Research, 28, 1–37.

[ele14063-bib-0009] Bjorkman, A.D. , Myers‐Smith, I.H. , Elmendorf, S.C. , Normand, S. , Rüger, N. , Beck, P.S.A. et al. (2018) Plant functional trait change across a warming tundra biome. Nature, 562, 57–62. 30258229 10.1038/s41586-018-0563-7

[ele14063-bib-0010] Blaus, A. , Reitalu, T. , Gerhold, P. , Hiiesalu, I. , Massante, J.C. & Veski, S. (2020) Modern pollen–plant diversity relationships inform Palaeoecological reconstructions of functional and phylogenetic diversity in calcareous fens. Frontiers in Ecology and Evolution, 8, 207.

[ele14063-bib-0011] Bogaard, A. , Fraser, R. , Heaton, T.H.E. , Wallace, M. , Vaiglova, P. , Charles, M. et al. (2013) Crop manuring and intensive land management by Europe's first farmers. Proceedings of the National Academy of Sciences, 110, 12589–12594. 10.1073/pnas.1305918110 PMC3732975 23858458

[ele14063-bib-0012] Bruelheide, H. , Dengler, J. , Purschke, O. , Lenoir, J. , Jimenez‐Alfaro, B. , Hennekens, S.M. et al. (2018) Global trait‐environment relationships of plant communities. Nature Ecology and Evolution, 2, 1906–1917. 30455437 10.1038/s41559-018-0699-8

[ele14063-bib-0013] Brussel, T. , Minckley, T.A. , Brewer, S.C. , Long, C.J. & Giesecke, T. (2018) Community‐level functional interactions with fire track long‐term structural development and fire adaptation. Journal of Vegetation Science, 29, 450–458.

[ele14063-bib-0014] Bunting, M.J. , Farrell, M. , Broström, A. , Hjelle, K.L. , Mazier, F. , Middleton, R. et al. (2013) Palynological perspectives on vegetation survey: a critical step for model‐based reconstruction of quaternary land cover. Quaternary Science Reviews, 82, 41–55.

[ele14063-bib-0015] Carvalho, F. , Brown, K.A. , Waller, M.P. , Bunting, M. , Jane Boom, A. & Leng, M.J. (2019) A method for reconstructing temporal changes in vegetation functional trait composition using Holocene pollen assemblages. PLoS One, 14, e0216698. 31141538 10.1371/journal.pone.0216698 PMC6541253

[ele14063-bib-0016] Cayuela, L. , Granzow‐de la Cerda, Í. , Albuquerque, F.S. & Golicher, D.J. (2012) Taxonstand: an R package for species names standardisation in vegetation databases. Methods in Ecology and Evolution, 3, 1078–1083.

[ele14063-bib-0017] Chamberlain, S. , Barve, V. , Mcglinn, D. , Oldoni, D. , Desmet, P. , Geffert, L. et al. (2021). Rgbif: Interface to the global biodiversity information facility.

[ele14063-bib-0018] Chen, S.H. (1988) Neue Untersuchungen über die spät‐und postglaziale Vegetationsgeschichte im Gebiet zwischen Harz und Leine (BRD). Flora, 181(3–4), 147–177.

[ele14063-bib-0019] Colombaroli, D. , Beckmann, M. , Knaap, W.O. , Curdy, P. & Tinner, W. (2013) Changes in biodiversity and vegetation composition in the central swiss Alps during the transition from pristine forest to first farming. Diversity and Distributions, 19, 157–170.

[ele14063-bib-0020] Connolly, A. (1999) The paleoecology of Clara bog, co. Offaly. In: Trinity college. Leinster, Ireland: University of Dublin.

[ele14063-bib-0021] Conti, G. & Díaz, S. (2013) Plant functional diversity and carbon storage – an empirical test in semi‐arid forest ecosystems. Journal of Ecology, 101, 18–28.

[ele14063-bib-0022] Cornwell, W.K. , Cornelissen, J.H. , Amatangelo, K. , Dorrepaal, E. , Eviner, V.T. , Godoy, O. et al. (2008) Plant species traits are the predominant control on litter decomposition rates within biomes worldwide. Ecology Letters, 11, 1065–1071. 18627410 10.1111/j.1461-0248.2008.01219.x

[ele14063-bib-0023] Cyprien, A.‐L. & Visset, L. (2001) Paleoenvironmental study of the Carquefou site (massif Armoricain, France) from the end of the sub‐boreal. Vegetation History and Archaeobotany, 10, 139–149.

[ele14063-bib-0024] Davies, A.L. (1999) High spatial resolution Holocene vegetation and land‐use history in west Glen Affric and Kintail. In: Department of Environmental Science. Stirling, UK: University of Stirling.

[ele14063-bib-0025] Davis, B.A. , Collins, P.M. & Kaplan, J.O. (2015) The age and post‐glacial development of the modern European vegetation: a plant functional approach based on pollen data. Vegetation History and Archaeobotany, 24, 303–317.

[ele14063-bib-0026] Dawson, A. , Paciorek, C.J. , McLachlan, J.S. , Goring, S. , Williams, J.W. & Jackson, S.T. (2016) Quantifying pollen‐vegetation relationships to reconstruct ancient forests using 19th‐century forest composition and pollen data. Quaternary Science Reviews, 137, 156–175.

[ele14063-bib-0027] De Beaulieu, J.‐L. , Leveau, P. , Miramont, C. , Palet, J. , Walsh, K. , Ricou, F. et al. (2003) Changements environnementaux postglaciaires et action de l'homme dans le bassin du Buëch et en Champasaur (Hautes‐Alpes, France). Premier bilan d'une étude pluridisciplinaire: Elsevier.

[ele14063-bib-0028] Denwood, M.J. (2016) Runjags: an R package providing Interface utilities, model templates, parallel computing methods and additional distributions for MCMC models in JAGS. Journal of Statistical Software, 71(9), 1–25.

[ele14063-bib-0029] Deyn, G.B. , Cornelissen, J.H.C. & Bardgett, R.D. (2008) Plant functional traits and soil carbon sequestration in contrasting biomes. Ecology Letters, 11, 516–531. 18279352 10.1111/j.1461-0248.2008.01164.x

[ele14063-bib-0030] Díaz, S. , Kattge, J. , Cornelissen, J.H.C. , Wright, I.J. , Lavorel, S. , Dray, S. et al. (2016) The global spectrum of plant form and function. Nature, 529, 167–171. 26700811 10.1038/nature16489

[ele14063-bib-0031] Douma, J.C. , de Haan, M.W.A. , Aerts, R. , Witte, J.P.M. & van Bodegom, P.M. (2012) Succession‐induced trait shifts across a wide range of NW European ecosystems are driven by light and modulated by initial abiotic conditions. Journal of Ecology, 100, 366–380.

[ele14063-bib-0032] Dreslerová, D. , Břízová, E. , Růžičková, E. & Zeman, A. (2004) Holocene environmental processes and alluvial archaeology in the middle Labe (Elbe) valley. In: Ancient landscape, settlement dynamics and non‐destructive archaeology. Praha: Academia, pp. 121–171.

[ele14063-bib-0033] Ellenberg, H. & Leuschner, C. (2010). Vegetation Mitteleuropas mit den Alpen: in ökologischer, dynamischer und historischer Sicht8104.

[ele14063-bib-0034] Ellis, E.C. , Gauthier, N. , Goldewijk, K.K. , Bird, R.B. , Boivin, N. , Díaz, S. et al. (2021) People have shaped most of terrestrial nature for at least 12,000 years. Proceedings of the National Academy of Sciences, 118, 118. 10.1073/pnas.2023483118 PMC8092386 33875599

[ele14063-bib-0035] Flantua, S.G.A. , Mottl, O. , Bhatta, K.P. , Felde, V.A. , Giesecke, T. , Goring, S. et al. (2021). Mottl et al. (2021, Science) Taxonomic harmonization tables for North America, Latin America, Europe, Asia, Africa. figshare.

[ele14063-bib-0036] Funk, J.L. , Larson, J.E. , Ames, G.M. , Butterfield, B.J. , Cavender‐Bares, J. , Firn, J. et al. (2017) Revisiting the holy grail: using plant functional traits to understand ecological processes. Biological Reviews, 92, 1156–1173. 27103505 10.1111/brv.12275

[ele14063-bib-0037] Fyfe, R.M. , Beaulieu, J.‐L.d. , Binney, H. , Bradshaw, R.H.W. , Brewer, S. , Flao, A. et al. (2009) The European pollen database: past efforts and current activities. Vegetation History and Archaeobotany, 18, 417–424.

[ele14063-bib-0038] Fyfe, R.M. , Brown, A.G. & Rippon, S.J. (2004) Characterising the late prehistoric,‘Romano‐British'and medieval landscape, and dating the emergence of a regionally distinct agricultural system in South West Britain. Journal of Archaeological Science, 31, 1699–1714.

[ele14063-bib-0039] Garnier, E. , Navas, M.‐L. & Grigulis, K. (2015) Plant traits and ecosystem properties. In: Plant functional diversity: organism traits, community structure, and ecosystem properties. Oxford: Oxford University Press, pp. 119–153.

[ele14063-bib-0040] Gauthier, E. (2001) Evolution de l'impact de l'homme sur la végétation du massif jurassien au cours des quatre derniers millénaires: nouvelles données palynologiques . Doctoral dissertation, Besançon.

[ele14063-bib-0041] GBIF . (2019). What is GBIF? Available at: https://www.gbif.org/what‐is‐gbif. Last accessed 11–6 2019.

[ele14063-bib-0042] Giesecke, T. , Wolters, S. , Leeuwen, J.F.N.v. , Knaap, P.W.O.v.d. , Leydet, M. & Brewer, S. (2019) Postglacial change of the floristic diversity gradient in Europe. Nature Communications, 10, 5422. 10.1038/s41467-019-13233-y PMC6882886 31780647

[ele14063-bib-0043] Gobet, E. , Tinner, W. , Hubschmid, P. , Jansen, I. , Wehrli, M. , Ammann, B. et al. (2000) Influence of human impact and bedrock differences on the vegetational history of the Insubrian southern Alps. Vegetation History and Archaeobotany, 9, 175–187.

[ele14063-bib-0044] Goring, S. , Dawson, A. , Simpson, G.L. , Ram, K. , Graham, R.W. , Grimm, E.C. et al. (2015). Neotoma: a programmatic Interface to the Neotoma Paleoecological database. 10.5334/oq.ab.

[ele14063-bib-0045] Grime, J.P. (1988) The CSR model of primary plant strategies—origins, implications and tests. In: Plant evolutionary biology. Dordrecht, The Netherlands: Springer, pp. 371–393.

[ele14063-bib-0046] Grime, J.P. (1998) Benefits of plant diversity to ecosystems: immediate, filter and founder effects. Journal of Ecology, 86, 902–910.

[ele14063-bib-0047] Hall, V.A. (1991) Detecting redeposited pollen in an Irish lake deposit. The Irish Naturalists' Journal, 23, 397–402.

[ele14063-bib-0048] Hawthorne, D. (2016) Quantifying fire regimes and their impact on the Irish landscape Doctoral dissertation, Trinity College Dublin.

[ele14063-bib-0049] Hevia, V. , Martin‐Lopez, B. , Palomo, S. , Garcia‐Llorente, M. , de Bello, F. & Gonzalez, J.A. (2017) Trait‐based approaches to analyze links between the drivers of change and ecosystem services: synthesizing existing evidence and future challenges. Ecology and Evolution, 7, 831–844. 28168020 10.1002/ece3.2692 PMC5288245

[ele14063-bib-0050] Hjelle, K.L. , Halvorsen, L.S. & Overland, A. (2010) Heathland development and relationship between humans and environment along the coast of western Norway through time. Quaternary International, 220, 133–146.

[ele14063-bib-0051] Hoffmann, T. , Schlummer, M. , Notebaert, B. , Verstraeten, G. & Korup, O. (2013) Carbon burial in soil sediments from Holocene agricultural erosion, Central Europe. Global Biogeochem Cy, 27, 828–835.

[ele14063-bib-0052] Houben, P. (2008) Scale linkage and contingency effects of field‐scale and hillslope‐scale controls of long‐term soil erosion: anthropogeomorphic sediment flux in agricultural loess watersheds of southern Germany. Geomorphology, 101, 172–191.

[ele14063-bib-0053] Jahns, S. (2007) Palynological investigations into the late Pleistocene and Holocene history of vegetation and settlement at the Löddigsee, Mecklenburg, Germany. Vegetation History and Archaeobotany, 16, 157–169.

[ele14063-bib-0054] Jeffers, E.S. , Nogué, S. & Willis, K.J. (2015) The role of palaeoecological records in assessing ecosystem services. Quaternary Science Reviews, 112, 17–32.

[ele14063-bib-0055] Joly, C. & Visset, L. (2009) Evolution of vegetation landscapes since the late Mesolithic on the French West Atlantic coast. Review of Palaeobotany and Palynology, 154, 124–179.

[ele14063-bib-0056] Joswig, J.S. , Wirth, C. , Schuman, M.C. , Kattge, J. , Reu, B. , Wright, I.J. et al. (2022) Climatic and soil factors explain the two‐dimensional spectrum of global plant trait variation. Nature Ecology and Evolution, 6, 36–50. 34949824 10.1038/s41559-021-01616-8 PMC8752441

[ele14063-bib-0057] Jouffroy‐Bapicot, I. , Vannière, B. , Gauthier, É. , Richard, H. , Monna, F. & Petit, C. (2013) 7000 years of vegetation history and land‐use changes in the Morvan Mountains (France): a regional synthesis. The Holocene, 23, 1888–1902.

[ele14063-bib-0058] Karger, D.N. , Nobis, M. P., Normand, S , Graham, C. H. & Zimmermann, N. E. (2020). CHELSA‐TraCE21k: downscaled transient temperature and precipitation data since the last glacial maximum. EnviDat. Available from: 10.16904/envidat.211

[ele14063-bib-0059] Kattge, J. , Bönisch, G. , Díaz, S. , Lavorel, S. , Prentice, I.C. , Leadley, P. et al. (2020) TRY plant trait database–enhanced coverage and open access. Global Change Biology, 26, 119–188. 31891233 10.1111/gcb.14904

[ele14063-bib-0060] Kaufman, D. , McKay, N. , Routson, C. , Erb, M. , Dätwyler, C. , Sommer, P.S. et al. (2020) Holocene global mean surface temperature, a multi‐method reconstruction approach. Scientific Data, 7, 201. 32606396 10.1038/s41597-020-0530-7 PMC7327079

[ele14063-bib-0061] Laliberte, E. & Tylianakis, J.M. (2012) Cascading effects of long‐term land‐use changes on plant traits and ecosystem functioning. Ecology, 93, 145–155. 22486095 10.1890/11-0338.1

[ele14063-bib-0062] Lavorel, S. & Grigulis, K. (2012) How fundamental plant functional trait relationships scale‐up to trade‐offs and synergies in ecosystem services. Journal of Ecology, 100, 128–140.

[ele14063-bib-0063] Leira, M. , Cole, E. & Mitchell, F. (2007) Peat erosion and atmospheric deposition impacts on an oligotrophic lake in eastern Ireland. Journal of Paleolimnology, 38, 49–71.

[ele14063-bib-0064] Litt, T. , Schoelzel, C. , Kuehl, N. & Brauer, A. (2009) Vegetation and climate history in the Westeifel volcanic field (Germany) during the past 11 000 years based on annually laminated lacustrine maar sediments. Boreas, 38, 679–690.

[ele14063-bib-0065] Lotter, A.F. (1999) Late‐glacial and Holocene vegetation history and dynamics as shown by pollen and plant macrofossil analyses in annually laminated sediments from Soppensee, Central Switzerland. Vegetation History and Archaeobotany, 8, 165–184.

[ele14063-bib-0066] Manning, K. , Colledge, S. , Crema, E. , Shennan, S. & Timpson, A. (2016) The cultural evolution of Neolithic Europe. EUROEVOL dataset 1: sites, phases and radiocarbon data. Journal of Open Archaeology Data, 5. Available from: 10.5334/joad.40

[ele14063-bib-0067] Marquer, L. , Gaillard, M.‐J. , Sugita, S. , Poska, A. , Trondman, A.‐K. , Mazier, F. et al. (2017) Quantifying the effects of land use and climate on Holocene vegetation in Europe. Quaternary Science Reviews, 171, 20–37.

[ele14063-bib-0068] Mazoyer, M. & Roudart, L. (2007) A history of world agriculture: from the neolithic age to the current crisis. London: Earthscan.

[ele14063-bib-0069] Mitchell, F. & Cooney, T. (2004) Vegetation history in the Killarney valley. Ross Island—mining, metal and society in early Ireland. Bronze Age Studies, 6, 481–493.

[ele14063-bib-0070] Moles, A.T. , Warton, D.I. , Warman, L. , Swenson, N.G. , Laffan, S.W. , Zanne, A.E. et al. (2009) Global patterns in plant height. Journal of Ecology, 97, 923–932.

[ele14063-bib-0071] Mottl, O. , Flantua, S.G.A. , Bhatta, K.P. , Felde, V.A. , Giesecke, T. , Goring, S. et al. (2021) Global acceleration in rates of vegetation change over the past 18,000 years. Science, 372, 860–864. 34016781 10.1126/science.abg1685

[ele14063-bib-0072] Nakagawa, T. (1998) Etudes palynologiques dans les Alpes Françaises centrales et méridionales: histoire de la végétation Tardiglaciaire et Holocène. Marseille, France: *Université d'Aix‐Marseille* .

[ele14063-bib-0073] Navas, M.L. (2012) Trait‐based approaches to unravelling the assembly of weed communities and their impact on agro‐ecosystem functioning. Weed Research, 52, 479–488.

[ele14063-bib-0074] Noël, H. , Garbolino, E. , Brauer, A. , Lallier‐Vergès, E. , De Beaulieu, J.‐L. & Disnar, J.‐R. (2001) Human impact and soil erosion during the last 5000 yrs as recorded in lacustrine sedimentary organic matter at lac d'Annecy, the French Alps. Journal of Paleolimnology, 25, 229–244.

[ele14063-bib-0075] Notebaert, B. , Verstraeten, G. , Rommens, T. , Vanmontfort, B. , Govers, G. & Poesen, J. (2009) Establishing a Holocene sediment budget for the river Dijle. Catena, 77, 150–163.

[ele14063-bib-0076] O'Carroll, E. (2012) Quantifying woodland resource usage in the Irish midlands using archaeological and palaeoecological techniques . Doctoral dissertation, Trinity College Dublin.

[ele14063-bib-0077] Parducci, L. , Bennett, K.D. , Ficetola, G.F. , Alsos, I.G. , Suyama, Y. , Wood, J.R. et al. (2017) Ancient plant DNA in lake sediments. New Phytologist, 214, 924–942. 28370025 10.1111/nph.14470

[ele14063-bib-0078] Parkes, H.M. & Mitchell, F.J. (2000) Vegetation history at Clonmacnoise, co. Offaly. In: Biology and environment: proceedings of the Royal Irish Academy. Dublin, Ireland: JSTOR, pp. 35–40.

[ele14063-bib-0079] Parnell, A. & Haslett, J. (2021) A simple monotone process with application to radiocarbon‐dated depth chronologies. Journal of the Royal Statistical Society: Series C (Applied Statistics), 57, 399–418.

[ele14063-bib-0080] Pierce, S. , Negreiros, D. , Cerabolini, B.E.L. , Kattge, J. , Diaz, S. , Kleyer, M. et al. (2017) A global method for calculating plant CSR ecological strategies applied across biomes world‐wide. Functional Ecology, 31, 444–457.

[ele14063-bib-0081] Plummer, M. (2004). JAGS: just another Gibbs sampler.

[ele14063-bib-0082] Plummer, M. , Best, N. , Cowles, K. & Vines, K. (2006) CODA: convergence diagnosis and output analysis for MCMC. R News, 6, 7–11.

[ele14063-bib-0083] Plunkett, G. (2009) Land‐use patterns and cultural change in the middle to late bronze age in Ireland: inferences from pollen records. Vegetation History and Archaeobotany, 18, 273–295.

[ele14063-bib-0084] Pokorny, P. (2005) Role of man in the development of Holocene vegetation in Central Bohemia. Preslia, 77, 113–128.

[ele14063-bib-0085] Pokorný, P. (2016) 29. Vrbka (Czech Republic): pollen record of secondary steppe vegetation development within the bronze age agricultural landscape. Grana, 55, 246–249.

[ele14063-bib-0086] R Core Team . (2021) R: a language and environment for statistical computing. Vienna, Austria: R Foundation for Statistical Computing.

[ele14063-bib-0087] Reich, P.B. (2014) The world‐wide ‘fast–slow’ plant economics spectrum: a traits manifesto. Journal of Ecology, 102, 275–301.

[ele14063-bib-0088] Reitalu, T. , Gerhold, P. , Poska, A. , Pärtel, M. , Väli, V. , Veski, S. et al. (2015) Novel insights into post‐glacial vegetation change: functional and phylogenetic diversity in pollen records. Journal of Vegetation Science, 26, 911–922.

[ele14063-bib-0089] Rey, F. , Gobet, E. , Schwörer, C. , Wey, O. , Hafner, A. & Tinner, W. (2019) Causes and mechanisms of synchronous succession trajectories in primeval central European mixed Fagus sylvatica forests. Journal of Ecology, 107, 1392–1408.

[ele14063-bib-0090] Roberts, N. (1998) Early Holocene adaptations. In: The Holocene. Oxford: Blackwell, pp. 99–101.

[ele14063-bib-0091] Roberts, N. , Fyfe, R.M. , Woodbridge, J. , Gaillard, M.J. , Davis, B.A.S. , Kaplan, J.O. et al. (2018) Europe's lost forests: a pollen‐based synthesis for the last 11,000 years. Scientific Reports, 8, 716. 29335417 10.1038/s41598-017-18646-7 PMC5768782

[ele14063-bib-0092] Rosch, M. (1990) Vegetationsgeschichtliche Untersuchungen im Durchenbergried in Siedlungsarchäologie im Alpenvorland II. Forschungen und Berichte zur Vor‐und Frühgeschichte in Baden‐Württemberg, 37, 9–64.

[ele14063-bib-0093] Rösch, M. (1993) Prehistoric land use as recorded in a lake‐shore core at Lake Constance. Vegetation History and Archaeobotany, 2, 213–232.

[ele14063-bib-0094] Rösch, M. & Lechterbeck, J. (2016) Seven millennia of human impact as reflected in a high resolution pollen profile from the profundal sediments of Litzelsee, Lake Constance region, Germany. Vegetation History and Archaeobotany, 25, 339–358.

[ele14063-bib-0095] Roucou, A. , Violle, C. , Fort, F. , Roumet, P. , Ecarnot, M. , Vile, D. et al. (2018) Shifts in plant functional strategies over the course of wheat domestication. Journal of Applied Ecology, 55, 25–37.

[ele14063-bib-0096] Rybníčková, E. , Hájková, P. & Rybníček, K. (2005) The origin and development of spring fen vegetation and ecosystems ‐ palaeogeobotanical results. In: Ecology and palaeoecology of spring fens of the West Carpathians. Olomouc, Czechia: Palacký University, pp. 29–62.

[ele14063-bib-0097] Schrodt, F. , Kattge, J. , Shan, H. , Fazayeli, F. , Joswig, J. , Banerjee, A. et al. (2015) BHPMF ‐ a hierarchical Bayesian approach to gap‐filling and trait prediction for macroecology and functional biogeography. Global Ecology and Biogeography, 24, 1510–1521.

[ele14063-bib-0098] Seppä, H. (2013) Encyclopedia of quaternary science (second edition). In: Paleobotany: Pollen Studies: Article Titles, Amsterdam, The Netherlands: Elsevier Science, pp. 794–804.

[ele14063-bib-0099] Siefert, A. , Violle, C. , Chalmandrier, L. , Albert, C.H. , Taudiere, A. , Fajardo, A. et al. (2015) A global meta‐analysis of the relative extent of intraspecific trait variation in plant communities. Ecology Letters, 18, 1406–1419. 26415616 10.1111/ele.12508

[ele14063-bib-0100] Simpson, G.L. (2018) Modelling Palaeoecological time series using generalised additive models. Frontiers in Ecology and Evolution, 6, 1–21.

[ele14063-bib-0101] Smith, A. & Goddard, I. (1991) A 12 500 year record of vegetational history at Sluggan bog, co. Antrim, N. Ireland (incorporating a pollen zone scheme for the non‐specialist). New Phytologist, 118, 167–187.

[ele14063-bib-0102] Stephens, L. , Fuller, D. , Boivin, N. , Rick, T. , Gauthier, N. , Kay, A. et al. (2019) Archaeological assessment reveals Earth's early transformation through land use. Science, 365, 897–902. 31467217 10.1126/science.aax1192

[ele14063-bib-0103] Svenning, J.‐C. , Fløjgaard, C. , Marske, K.A. , Nógues‐Bravo, D. & Normand, S. (2011) Applications of species distribution modeling to paleobiology. Quaternary Science Reviews, 30, 2930–2947.

[ele14063-bib-0104] Svenning, J.C. (2002) A review of natural vegetation openness in North‐Western Europe. Biological Conservation, 104, 133–148.

[ele14063-bib-0105] The Plant List . (2013). Version 1.1. Available at: http://www.theplantlist.org/. Last accessed 11 2020.

[ele14063-bib-0106] Thomas, H.J.D. , Myers‐Smith, I.H. , Bjorkman, A.D. , Elmendorf, S.C. , Blok, D. , Cornelissen, J.H.C. et al. (2019) Traditional plant functional groups explain variation in economic but not size‐related traits across the tundra biome. Global Ecology and Biogeography, 28, 78–95. 31007605 10.1111/geb.12783 PMC6472633

[ele14063-bib-0107] Tinner, W. , Conedera, M. , Ammann, B. , Gaggeler, H.W. , Gedye, S. , Jones, R. et al. (1998) Pollen and charcoal in lake sediments compared with historically documented forest fires in southern Switzerland since AD 1920. The Holocene, 8, 31–42.

[ele14063-bib-0108] Trondman, A.K. , Gaillard, M.J. , Mazier, F. , Sugita, S. , Fyfe, R. , Nielsen, A.B. et al. (2015) Pollen‐based quantitative reconstructions of Holocene regional vegetation cover (plant‐functional types and land‐cover types) in Europe suitable for climate modelling. Global Change Biology, 21, 676–697. 25204435 10.1111/gcb.12737

[ele14063-bib-0109] van der Knaap, W.O. , van Leeuwen, J.F. , Fankhauser, A. & Ammann, B. (2000) Palynostratigraphy of the last centuries in Switzerland based on 23 lake and mire deposits: chronostratigraphic pollen markers, regional patterns, and local histories. Review of Palaeobotany and Palynology, 108, 85–142. 10.1016/s0034-6667(01)00049-5 11389919

[ele14063-bib-0110] van der Sande, M.T. , Gosling, W. , Correa‐Metrio, A. , Prado‐Junior, J. , Poorter, L. , Oliveira, R.S. et al. (2019) A 7000‐year history of changing plant trait composition in an Amazonian landscape; the role of humans and climate. Ecology Letters, 22, 925–935. 30883016 10.1111/ele.13251 PMC6850629

[ele14063-bib-0111] Volaire, F. , Gleason, S.M. & Delzon, S. (2020) What do you mean “functional” in ecology? Patterns versus processes. Ecology and Evolution, 10, 11875–11885. 33209257 10.1002/ece3.6781 PMC7663066

[ele14063-bib-0112] Waller, M. (1987) The Flandrian vegetational history and environmental development of the Brede and Pannel valleys. East Sussex: University of East London.

[ele14063-bib-0113] Waller, M. & Marlow, A. (1994) Flandrian vegetational history of south‐eastern England. Stratigraphy of the Brede valley and pollen data from Brede bridge. New Phytologist, 126, 369–392.

[ele14063-bib-0114] Webb, N.R. (1998) The traditional management of European heathlands. Journal of Applied Ecology, 35, 987–990.

[ele14063-bib-0115] Welten, M. (1982) Pollenanalytische Untersuchungen zur Vegetationsgeschichte des Schweizerischen Nationalparks. E. Ergebnisse der wissenschaftlichen Untersuchungen im Schweizerischen Nationalpark, 16, 1–43.

[ele14063-bib-0116] Westoby, M. , Falster, D.S. , Moles, A.T. , Vesk, P.A. & Wright, I.J. (2002) Plant ecological strategies: some leading dimensions of variation between species. Ecology System, 33, 125–159.

[ele14063-bib-0117] Wieczorek, M. & Herzschuh, U. (2020) Compilation of relative pollen productivity (RPP) estimates and taxonomically harmonised RPP datasets for single continents and northern hemisphere extratropics. Earth System Science Data, 12, 3515–3528.

[ele14063-bib-0118] Williams, J.W. , Grimm, E.C. , Blois, J.L. , Charles, D.F. , Davis, E.B. , Goring, S.J. et al. (2018) The Neotoma paleoecology database, a multiproxy, international, community‐curated data resource. Quaternary Research, 89, 156–177.

[ele14063-bib-0119] Wood, S.N. (2016) Just another gibbs additive modeller: interfacing JAGS and mgcv. arXiv preprint arXiv:1602.02539. Available from: 10.48550/arXiv.1602.02539

[ele14063-bib-0120] Wood, S.N. (2017) Generalized additive models: an introduction with R, 2nd edition. New York, NY: Chapman and Hall/CRC.

[ele14063-bib-0121] Woodbridge, J. , Fyfe, R. , Smith, D. , Pelling, R. , Vareilles, A. , Batchelor, R. et al. (2020) What drives biodiversity patterns? Using long‐term multidisciplinary data to discern centennial‐scale change. Journal of Ecology, 109, 1396–1410.

[ele14063-bib-0122] Wright, I.J. , Dong, N. , Maire, V. , Prentice, I.C. , Westoby, M. , Díaz, S. et al. (2017) Global climatic drivers of leaf size. Science, 357, 917–921. 28860384 10.1126/science.aal4760

[ele14063-bib-0123] Wright, I.J. , Reich, P.B. , Cornelissen, J.H.C. , Falster, D.S. , Garnier, E. , Hikosaka, K. et al. (2005) Assessing the generality of global leaf trait relationships. New Phytologist, 166, 485–496. 15819912 10.1111/j.1469-8137.2005.01349.x

[ele14063-bib-0124] Wright, I.J. , Reich, P.B. , Westoby, M. , Ackerly, D.D. , Baruch, Z. , Bongers, F. et al. (2004) The worldwide leaf economics spectrum. Nature, 428, 821–827. 15103368 10.1038/nature02403

[ele14063-bib-0125] Zizka, A. , Silvestro, D. , Andermann, T. , Azevedo, J. , Duarte Ritter, C. , Edler, D. et al. (2019) CoordinateCleaner: standardized cleaning of occurrence records from biological collection databases. Methods in Ecology and Evolution, 10, 744–751.

[ele14063-bib-0126] Zuazo, V.H.D. , & Pleguezuelo, C.R.R. (2009). Soil‐erosion and runoff prevention by plant covers: a review. In: Sustainable agriculture. Dordrecht: Springer.

