# Supplementary material for: Pollen‐based reconstruction reveals the impact of the onset of agriculture on plant functional trait composition
Source: Ecol Lett. 2022 Jul 11;25(9):1937–51. doi: 10.1111/ele.14063 (PMC9544184; doi:10.1111/ele.14063)
Supplement: Supplementary file 1 — Data S1 [file ELE-25-1937-s001.docx]

Table of Contents

[Appendix S1: Search terms 2](#_Toc97213869)

[Appendix S2: Site characteristics and references 3](#_Toc97213870)

[Appendix S3: Characteristics of the pollen taxa 17](#_Toc97213871)

[Appendix S4: JAGS code 19](#_Toc97213872)

[Appendix S5: Comparison between univariate and multivariate likelihood for CWM calculation 23](#_Toc97213873)

[Appendix S6: PCA plots 25](#_Toc97213874)

[Appendix S7: Evaluation of GAMs 29](#_Toc97213875)

[Appendix S8: Temperature data 38](#_Toc97213876)

[Appendix S9: Correlogram CWM trait values 39](#_Toc97213877)

[Appendix S10: Taxon level trait estimates 41](#_Toc97213878)

[References 45](#_Toc97213879)

# Appendix S1: Search terms

Search terms for the structured search in Web of Science

TS= (agricultur* OR cultivat* OR farm* OR agrarian OR agro*)

AND

TS = (Holocene OR prehistor* OR pal*eo* OR Neolithic OR Mesolithic OR "Bronze Age" OR "Iron Age" OR Roman OR "middle ages" OR Medieval OR BP OR AD OR BC OR millen*)

AND

TS = (Europe* OR Netherlands OR Dutch OR Belgium OR Belgian OR German* OR "United Kingdom" OR "Great Britain" OR British OR Ireland OR Irish OR "British Isles" OR France OR French OR Switzerland OR Swiss OR Austria* OR Sweden OR Swedish OR Denmark OR Danish OR "Czech Republic" OR Czech* OR Scandinavia)

# Appendix S2: Site characteristics and references

.
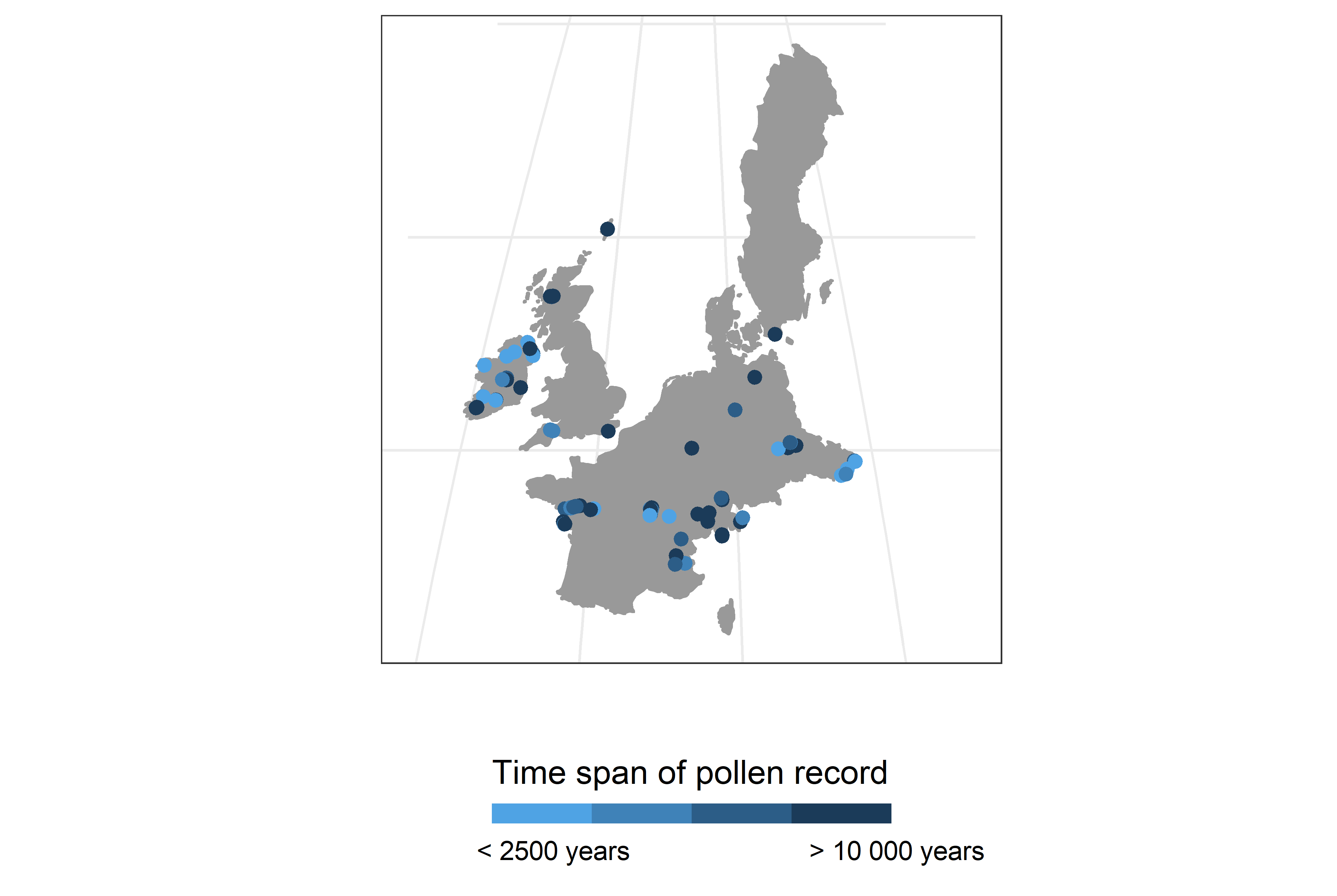


Figure 1 Location of the 78 pollen records that were used in this study. Sites are colored by the time span of the pollen record.

Table 1 Site characteristics and references

| Site name | Elevation  (m a.s.l.) | Site type | Size | Citation |
| --- | --- | --- | --- | --- |
| Ancenis | 5 | Marsh | - | Cyprien, A.L. 2001. Chronologie de l'interaction de l'homme et du milieu dans l'espace central et aval de la Loire (Ouest de la France). Doctoral dissertation. Université de Nantes, Nantes, France.  Cyprien, A.L., L. Visset, and N. Carcaud. 2004. Evolution of vegetation landscapes during the Holocene in the central and downstream Loire basin (Western France). Vegetation History and Archaeobotany 13(3):181-196.  Pastore, R.T. 1987. Fishermen, furriers, and Beothuks: the economy of extinction. Man in the Northeast 33:47-62. |
| Ballinderry | 80 | Lake | 1.3  ha | OCarroll, E. 2012. Quantifying woodland resource usage in the Irish midlands using archaeological and palaeoecological techniques. Doctoral dissertation. University of Dublin, Trinity College, Dublin, Ireland. |
| Ballynahatty bog | 36 | Lake | 0.2  ha | Plunkett, G., F. Carroll, B. Hartwell, N.J. Whitehouse, and P.J. Reimer. 2008. Vegetation history at the multi-period prehistoric complex at Ballynahatty, Co. Down, Northern Ireland. Journal of Archaeological Science 35:181-190. |
| Bjärsjöholmssjön | 50 | Ancient lake | - | Göransson, H. 1991. Vegetation and man around Lake Bjärsjöholmssjön during prehistoric time. Lundqua Report 31.  Berglund, B.E., M.J. Gaillard-Lemdahl, and H. Göransson. 1991. The Bjäresjö area. Pages 167-174 in B.E. Berglund, editor. The cultural landscape during 6000 years in southern Sweden - the Ystad project. Ecological Bulletins 41. |
| Borheen Lough | 476 | Lake | 2.45  ha | Hawthorne, D., & Mitchell, F. J. (2016). Identifying past fire regimes throughout the Holocene in Ireland using new and established methods of charcoal analysis. Quaternary Science Reviews, 137, 45-53. |
| Brede Bridge | 2 | Floodplain mire | - | Waller, M.P. 1993. Flandrian vegetational history of south-eastern England. Stratigraphy of the Brede valley and pollen data from Brede Bridge. New Phytologist 126:369-392. |
| Camban |  | Peat basin | - | Davies A (1999) High spatial resolution Holocene vegetation and land-use history in west Glen Affric and Kintail. University of Stirling |

Table 1 (continued)

| Site name | Elevation  (m a.s.l.) | Site type | Size | Citation |
| --- | --- | --- | --- | --- |
| Carquefou | 11 | Bog | - | Cyprien, A.L. 2001. Chronologie de l'interaction de l'homme et du milieu dans l'espace central et aval de la Loire (Ouest de la France).. Doctoral dissertation. Université de Nantes, Nantes, France.  Cyprien, A.L., and L. Visset. 2001. Paleoenvironmental study of the Carquefou site (Massif Armoricain, France) from the end of the Sub-boreal. Vegetation History and Archaeobotany 10(3):139-149.  Visset, L., A.L. Cyprien, A. Ouguerram, D. Barbier, and J. Bernard. 2004. Les indices polliniques d'anthropisation précoce dans l'Ouest de la France: le cas de Cerealia, Fagopyrum et Juglans. Annales Littéraires, Série Environnement, Société et Archéologie 777(7):69-79. |
| Carrach Mor |  | Peat basin | - | Davies A (1999) High spatial resolution Holocene vegetation and land-use history in west Glen Affric and Kintail. University of Stirling |
| Champ Gazon | 585 | Blanket mire | - | Jouffroy-Bapicot, I. 2010. Evolution de la végétation du massif du Morvan (Bourgogne - France) depuis la dernière glaciation à partir de l'analyse pollinique. Variations climatiques et impact des activités anthropiques.. Doctoral dissertation. Université de Franche-Comté, Besançon, France. Jouffroy-Bapicot, I., B. Vannière, E. Gauthier, H. Richard, F. Monna, and C. Petit. 2013. 7000 years of vegetation history and land-use changes in the Morvan Mountains (France): A regional synthesis. The Holocene 23(12):1888-1902. |
| Changeon | 28 | Riverine | - | Cyprien, A.L. 2001. Chronologie de l'interaction de l'homme et du milieu dans l'espace central et aval de la Loire (Ouest de la France). Doctoral dissertation. Université de Nantes, Nantes, France. Cyprien, A.L., L. Visset, and N. Carcaud. 2004. Evolution of vegetation landscapes during the Holocene in the central and downstream Loire basin (Western France). Vegetation History and Archaeobotany 13(3):181-196. |
| Clara Bog | 59 | Raised bog | - | Connolly, A. 1999. The Paleoecology of Clara Bog, Co. Offaly. Doctoral dissertation. Trinity College, University of Dublin, Dublin, Leinster, Ireland. |
| Claraghmore | 78 | Raised bog | 51  ha | Plunkett, G. 2009. Land-use patterns and cultural change in the Middle to Late Bronze Age Ireland: inferences from pollen records. Vegetation History Archaeobotany 18:273-295. |
| Cordemais | 5 | Marsh | - | Cyprien, A.L. 2001. Chronologie de l'interaction de l'homme et du milieu dans l'espace central et aval de la Loire (Ouest de la France). Doctoral dissertation. Université de Nantes, Nantes, France. Visset, L., A.L. Cyprien, A. Ouguerram, D. Barbier, and J. Bernard. 2004. Les indices polliniques d'anthropisation précoce dans l'Ouest de la France: le cas de Cerealia, Fagopyrum et Juglans. Annales Littéraires, Série Environnement, Société et Archéologie 777(7):69-79. |

Table 1 (continued).

| Site name | Elevation (m a.s.l.) | Site type | Size | Citation |
| --- | --- | --- | --- | --- |
| Cuckoo | 32 | Lake | 0.15  ha | Hawthorne, D. 2015. Quantifying fire regimes and their impact on the Irish Landscape. Doctoral dissertation. Trinity College Dublin, Dublin, Leinster, Ireland. |
| Dallican Water | 56 | Lake | 1  ha | Bennett, K., S. Boreham, M.J. Sharp, and V.R. Switsur. 1992. Holocene history of environment, vegetation and human settlement on Catta Ness, Lunnasting, Shetland. Journal of Ecology 80:241-273. https://www.jstor.org/stable/2261010. [DOI: 10.2307/2261010] |
| Diheen | 554 | Lake | 0.79  ha | Hawthorne, D., & Mitchell, F. J. (2016). Identifying past fire regimes throughout the Holocene in Ireland using new and established methods of charcoal analysis. Quaternary Science Reviews, 137, 45-53. |
| Durchenbergried | 432 | Fen | 3  ha | Rösch, M. 1990. Vegetationsgeschichtliche Untersuchungen im Durchenbergried. Pages 9-64 in Siedlungsarchäologie im Alpenvorland II. Forschungen und Berichte zur Vor- und Frühgeschichte in Baden-Württemberg 37. Theiss, Stuttgart. Rösch, M. 1986. Zwei Moore im westlichen Bodenseegebiet als Zeugen prähistorischer Landschaftsveränderung. Telma 16:83-111. |
| Ecours | 4 | Riverine | - | Joly, C. 2006. Histoire de la végétation dans l'espace centre-ouest atlantique (France): relations Sociétés/Végétation et évolution du trait de côte depuis le Mésolithique récent-final. Doctoral dissertation. Université de Nantes, Nantes, France. Joly, C., and L. Visset. 2009. Evolution of vegetation landscapes since the Late Mesolithic on the French West Atlantic coast. Review of Palaeobotany and Palynology 154:124-179. |
| Etang Bouquin | 540 | Bog | - | Jouffroy-Bapicot, I. 2010. Evolution de la végétation du massif du Morvan (Bourgogne - France) depuis la dernière glaciation à partir de l'analyse pollinique. Variations climatiques et impact des activités anthropiques.. Doctoral dissertation. Université de Franche-Comté, Besançon, France. Jouffroy-Bapicot, I., B. Vannière, E. Gauthier, H. Richard, F. Monna, and C. Petit. 2013. 7000 years of vegetation history and land-use changes in the Morvan Mountains (France): A regional synthesis. The Holocene 23(12):1888-1902. |

Table 1 (continued).

| Site name | Elevation  (m a.s.l.) | Site type | Site | Citation |
| --- | --- | --- | --- | --- |
| Fangeas | 2000 | Marsh | 60m  max. | de Beaulieu, J.L., P. Leveau, C. Miramont, J.M. Palet- Martinez, K. Walsh, M. Court-Picon, F. Ricou, M. Segard, O. Sivan, V. Andrieu-Ponel, M. Badura, G. Bertucchi, C. Bouterin, A. Durand, J.L. Edouard, M. Lavoie, A. Morin, F. Mocci, P. Ponel, A. Pothin, V. Py, B. Talon, S. Tzortzis, R. Bonet, P. Columeau, H. Cortot, and D. Garcia. 2003. Changements environnementaux postglaciaires et action de l'homme dans le bassin du Buëch et en Champsaur (Hautes- Alpes, France). Premier bilan d'une étude pluridisciplinaire. Pages 93-101 in E.P. Collection Environnement, editor. Des Milieux et des Hommes: Fragments d'Histoires Croisées. France. Walsh, K., and F. Mocci. 2003. 9000 ans d'occupation du sol en moyenne et haute montagne: la vallée de Freissinières dans le Parc national des Ecrins (Freissinières, Hautes- Alpes). Archéologie du Midi Médiéval 21:185-198. |
| Garry Bog | 50 | Raised bog | 155  ha | Plunkett, G. 2009. Land-use patterns and cultural change in the Middle to Late Bronze Age Ireland: inferences from pollen records. Vegetation History Archaeobotany 18:273-295. |
| Glen West | 90 | Raised bog | 152  ha | Plunkett, G. 2009. Land-use patterns and cultural change in the Middle to Late Bronze Age Ireland: inferences from pollen records. Vegetation History Archaeobotany 18:273-295. Plunkett, G., J.R. Pilcher, F.G. McCormac, and V.A. Hall. 2004. New dates for first millennium BC tephra isochrones in Ireland. The Holocene 14(5):780-786. |
| Hares Down | 255 | Valley mire | 15 x 200  m | Fyfe, R.M., A.G. Brown, and S.J. Rippon. 2004. Characterising the late prehistoric, "Romano-British" and medieval landscape, and dating the emergence of a regionally distinct agricultural system in South West Britain. Journal of Archaeological Science 31(12):1699-1714. |
| Holzmaar | 425 | Volcanic lake | 325 m Ø | Brauer, A., T. Litt, J.F.W. Negendank, and B. Zolitschka. 2001. Lateglacial varve chronology and biostratigraphy of lakes Holzmaar and Meerfelder Maar, Germany. Boreas 30:83-88. Litt, T., and M. Stebich. 1999. Bio- and chronostratigraphy of the lateglacial in the Eifel region, Germany. Quaternary International 61:5-16. Litt, T., C. Schölzel, N. Kühl, and A. Brauer. 2009. Vegetation and climate history in the Westeifel Volcanic Field (Germany) during the past 11 000 years based on annually laminated lacustrine maar sediments. Boreas 38:679-690. Litt, T., M. Früchtl, B. Kubitz, and M. Stebich. 1997. Jungquartäre Floren in den Eifelmaaren. Terra Nostra 1997(7):54-62. Notes: Exkursionsführer zur 67. Jahrestagung der Paläontologischen Gesellschaft |

Table 1 (continued).

| Site name | Elevation  (m a.s.l.) | Site type | Size | Citation |
| --- | --- | --- | --- | --- |
| Horní Lomná | 615 | Spring fen | 0.04  ha | Rybníčková, E., P. Hájková, and K. Rybníček. 2005. The origin and development of spring fen vegetation and ecosystems - palaeogeobotanical results. Pages 29-57 in A. Poulíčková, M. Hájek, and K. Rybníček, editors. Ecology and palaeoecology of spring fens of the West Carpathians. Palacký University Olomouc, Academy of Sciences of the Czech Republic, Masaryk University Brno, Olomouc. Rybníček, K., and E. Rybníčková. 2008. Upper Holocene dry land vegetation in the Moravian–Slovakian borderland (Czech and Slovak Republics). Vegetation History and Archaeobotany 17(6):701–711. |
| Hornstaad-Hörnle | 385 | Lake | - | Rösch, M. 1992. Human impact as registered in the pollen record: some results from the western Lake Constance region, Southern Germany. Vegetation History and Archaeobotany 1:101-109. Rösch, M. 1993. Prehistoric land use as recorded in a lake-shore core at Lake Constance. Vegetation History and Archaeobotany 2:213-232. Rösch, M. 1997. Holocene sediment accumulation in the shallow water zone of Lower Lake Constance. Archiv für Hydrobiologie Supplement 107(4):541-562. |
| Il Fuorn | 1805 | Peat bog | 10 x 10 m | Welten, M. 1982. Pollenanalytische Untersuchungen zur Vegetationsgeschichte des Schweizerischen Nationalparks. Ergebnisse der wissenschaftlichen Untersuchungen im Schweizerischen Nationalpark 16:1-43. |
| Jaunay | 1 | Riverine | 1 | Joly, C. 2006. Histoire de la végétation dans l'espace centre-ouest atlantique (France) : relations Sociétés Végétation et évolution du trait de côte depuis le Mésolithique récent-final.. Doctoral dissertation. Université de Nantes, Nantes, France. Joly, C., and L. Visset. 2009. Evolution of vegetation landscapes since the Late Mesolithic on the French West Atlantic coast. Review of Palaeobotany and Palynology 154:124-179. |
| Kalven | 163 | Lake | - | Björck, S., and P. Möller. 1987. Late Weichselian environmental history in southeastern Sweden during the deglaciation of the Scandinavian ice sheet. Quaternary Research 28:1-37. [DOI: https://doi.org/10.1016/0033-5894(87)90030-5] |
| Kelly's Lough | 585 | Lake | 3 | Leira, M., E.E. Cole, and F.J.G. Mitchell. 2007. Peat erosion and atmospheric deposition impacts on an oligotrophic lake in eastern Ireland. Journal of Paleolimnology 38(1):49-71. [DOI: 10.1007/s10933-006-9060-3] |

Table 1 (continued).

| Site name | Elevation  (m a.s.l.) | Site type | Size | Citation | |
| --- | --- | --- | --- | --- | --- |
| Královec | 599 | Spring fen | 0.025 ha | Rybníčková, E., P. Hájková, and K. Rybníček. 2005. The origin and development of spring fen vegetation and ecosystems - palaeogeobotanical results. Pages 29-57 in A. Poulíčková, M. Hájek, and K. Rybníček, editors. Ecology and palaeoecology of spring fens of the West Carpathians. Palacký University Olomouc, Academy of Sciences of the Czech Republic, Masaryk University Brno, Olomouc. Rybníček, K., and E. Rybníčková. 2008. Upper Holocene dry land vegetation in the Moravian–Slovakian borderland (Czech and Slovak Republics). Vegetation History and Archaeobotany 17(6):701–711. |  |
| Kubriková | 790 | Spring fen | 0.05 ha | Rybníčková, E., P. Hájková, and K. Rybníček. 2005. The origin and development of spring fen vegetation and ecosystems - palaeogeobotanical results. Pages 29-57 in A. Poulíčková, M. Hájek, and K. Rybníček, editors. Ecology and palaeoecology of spring fens of the West Carpathians. Palacký University Olomouc, Academy of Sciences of the Czech Republic, Masaryk University Brno, Olomouc. Rybníček, K., and E. Rybníčková. 2008. Upper Holocene dry land vegetation in the Moravian–Slovakian borderland (Czech and Slovak Republics). Vegetation History and Archaeobotany 17(6):701–711. | |
| Lac de Praver | 1170 | Lake | - | Nakagawa, T. 1998. Etudes palynologiques dans les Alpes Françaises centrales et méridionales: histoire de la végétation Tardiglaciaire et Holocène. Doctoral dissertation. Université d'Aix-Marseille, Marseille, France. | |
| Lac du Lauzon | 1980 | Lake | - | Argant, J., and A. Argant. 2000. Mise en évidence de l'occupation ancienne d'un site d'altitude: analyse pollinique du lac de Lauzon (Drôme). Géologie Alpine 31:61-71. | |
| Lago di Muzzano | 337 | Lake | 22 ha | Gobet, E., W. Tinner, P. Hubschmid, I. Jansen, M. Wehrli, B. Ammann, and L. Wick. 2000. Influence of human impact and bedrock differences on the vegetational history of the Insubrian Southern Alps. Vegetation History and Archaeobotany 9:175-187. Tinner, W., P. Hubschmid, M. Wehrli, B. Ammann, and M. Conedera. 1999. Long-term forest fire ecology and dynamics in southern Switzerland. Journal of Ecology 87(2):273-289. [DOI: 10.1046/j.1365-2745.1999.00346.x] | |
| Lago di Origlio | 416 | Lake | 8 ha | Tinner, W., M. Conedera, B. Ammann, H.W. Gaggeler, S. Gedye, R. Jones, and B. Sagesser. 1998. Pollen and charcoal in lake sediments compared with historically documented forest fires in southern Switzerland since AD 1920. The Holocene 8(1):31-42. | |

Table 1 (continued).

| Site name | Elevation  (m a.s.l.) | Site type | Site | Citation |
| --- | --- | --- | --- | --- |
| Lake of Annecy | 447 | Lake | 625 ha | Noël, H. 2001. Caractérisation et calibration des flux organiques sédimentaires dérivant du bassin versant et de la production aquatique (Annecy, Le Petit Lac) -Rôles respectifs de l'Homme et du Climat surl'évolution des flux organiques au cours des 6000 dernières années.. Doctoral dissertation. Université d'Orléans, Orléans, France. Dearing, J.A., Y. Hu, P. Doody, P.A. James, and A. Brauer. 2001. Preliminary reconstruction of sediment-source linkages for the past 6000 yrs at the Petit Lac d'Annecy, France, based on mineral magnetic data. Journal of Paleolimnology 25:245-258. Noël, H., E. Garbolino, A. Brauer, E. Lallier-Vergès, J.L. de Beaulieu, and J.R. Disnar. 2001. Human impact and soil erosion during the last 5000 yrs as recorded in lacustrine sedimentary organic matter at Lac d'Annecy, the French Alps. Journal of Paleolimnology 25:229-244. |
| Le Grand Montarnu | 771 | Bog | - | Jouffroy-Bapicot, I. 2010. Evolution de la végétation du massif du Morvan (Bourgogne - France) depuis la dernière glaciation à partir de l'analyse pollinique. Variations climatiques et impact des activités anthropiques.. Doctoral dissertation. Université de Franche-Comté, Besançon, France. Jouffroy-Bapicot, I., B. Vannière, E. Gauthier, H. Richard, F. Monna, and C. Petit. 2013. 7000 years of vegetation history and land-use changes in the Morvan Mountains (France): A regional synthesis. The Holocene 23(12):1888-1902. |
| Le Vernay | 595 | Bog | - | Jouffroy-Bapicot, I. 2010. Evolution de la végétation du massif du Morvan (Bourgogne - France) depuis la dernière glaciation à partir de l'analyse pollinique. Variations climatiques et impact des activités anthropiques.. Doctoral dissertation. Université de Franche-Comté, Besançon, France. Jouffroy-Bapicot, I., B. Vannière, E. Gauthier, H. Richard, F. Monna, and C. Petit. 2013. 7000 years of vegetation history and land-use changes in the Morvan Mountains (France): A regional synthesis. The Holocene 23(12):1888-1902. |
| Le Verny des Brûlons | 565 | Bog | - | Jouffroy-Bapicot, I. 2010. Evolution de la végétation du massif du Morvan (Bourgogne - France) depuis la dernière glaciation à partir de l'analyse pollinique. Variations climatiques et impact des activités anthropiques.. Doctoral dissertation. Université de Franche-Comté, Besançon, France. Jouffroy-Bapicot, I., B. Vannière, E. Gauthier, H. Richard, F. Monna, and C. Petit. 2013. 7000 years of vegetation history and land-use changes in the Morvan Mountains (France): A regional synthesis. The Holocene 23(12):1888-1902. |

Table 1 (continued).

| Site name | Elevation  (m a.s.l.) | Site type | Size | Citation |
| --- | --- | --- | --- | --- |
| Litzelsee | 413 | Lake | 1.3 | Rösch, M., and J. Lechterbeck. 2016. Seven Millennia of human impact as reflected in a high resolution pollen profile from the profundal sediments of Litzelsee, Lake Constance region, Germany. Vegetation History and Archaeobotany 25:339-358. |
| Lobbs Bog | 243 | Valley mire | - | Fyfe, R.M., A.G. Brown, and S.J. Rippon. 2004. Characterising the late prehistoric, "Romano-British" and medieval landscape, and dating the emergence of a regionally distinct agricultural system in South West Britain. Journal of Archaeological Science 31(12):1699-1714. |
| Lobsigensee | 514 | Lake | 8 ha | Ammann, B. 1985. Lobsigensee - Late Glacial and Holocene environments of a lake on the central Swiss plateau. Dissertationes Botanicae 87:127-134. |
| Löddigsee | 43 | Lake | 150  ha | Jahns, S. 2007. Palynological inverstigations into the Late Pleistocene and Holocene history of vegetation and settlement at the Löddigsee, Mecklenburg, Germany. Vegetation History Archaeobotany 16:157-169. |
| Logné | 4 | Bog | 120 ha | Barbier, D., and L. Visset. 1997. Logné, a peat bog of European ecological interest in the Massif Armorican, western France: bog development, vegetation and land-use history. Vegetation History and Archaeobotany 6:69-77. |
| Lough Henney | 25 | Lake | 9.7  ha | Hall, V.A. 1989. A comparative study of the palynology and regional history of some sites in the north of Ireland. Doctoral dissertation. Queen's University, Belfast, Northern Ireland, UK. Hall, V.A. 1991. Detecting redeposited pollen in an Irish lake deposit. Irish Naturalists' Journal 23(10):397-402. https://www.jstor.org/stable/25539592. Hall, V.A., S.J. McVicker, and J.R. Pilcher. 1994. Tephra-linked landscape history around 2310 BC of some sites in Counties Antrim and Down. Proceedings of the Royal Irish Academy, Biology and Environment 94B(3):245-253. https://www.jstor.org/stable/20499942. |
| Machová | 498 | Spring fen | 0.05 ha | Rybníčková, E., P. Hájková, and K. Rybníček. 2005. The origin and development of spring fen vegetation and ecosystems - palaeogeobotanical results. Pages 29-57 in A. Poulíčková, M. Hájek, and K. Rybníček, editors. Ecology and palaeoecology of spring fens of the West Carpathians. Palacký University Olomouc, Academy of Sciences of the Czech Republic, Masaryk University Brno, Olomouc. Rybníček, K., and E. Rybníčková. 2008. Upper Holocene dry land vegetation in the Moravian–Slovakian borderland (Czech and Slovak Republics). Vegetation History and Archaeobotany 17(6):701–711. |

Table 1 (continued).

| Site name | Elevation  (m a.s.l.) | Site type | Size | Citation |
| --- | --- | --- | --- | --- |
| Marais de Champtocé | 19 | Marsh | - | Cyprien, A.L. 2001. Chronologie de l'interaction de l'homme et du milieu dans l'espace central et aval de la Loire (Ouest de la France). Doctoral dissertation. Université de Nantes, Nantes, France. Cyprien, A.L., L. Visset, and N. Carcaud. 2004. Evolution of vegetation landscapes during the Holocene in the central and downstream Loire basin (Western France). Vegetation History and Archaeobotany 13(3):181-196. Visset, L., A.L. Cyprien, A. Ouguerram, D. Barbier, and J. Bernard. 2004. Les indices polliniques d'anthropisation précoce dans l'Ouest de la France: le cas de Cerealia, Fagopyrum et Juglans. Annales Littéraires, Série Environnement, Société et Archéologie 777(7):69-79. |
| Marais de Méron | 6 | Marsh | - | Cyprien, A.L. 2001. Chronologie de l'interaction de l'homme et du milieu dans l'espace central et aval de la Loire (Ouest de la France). Doctoral dissertation. Université de Nantes, Nantes, France. Cyprien, A.L., L. Visset, and N. Carcaud. 2004. Evolution of vegetation landscapes during the Holocene in the central and downstream Loire basin (Western France). Vegetation History and Archaeobotany 13(3):181-196. |
| Marais de Munet | 28 | Marsh | - | Cyprien, A.L. 2001. Chronologie de l'interaction de l'homme et du milieu dans l'espace central et aval de la Loire (Ouest de la France). Doctoral dissertation. Université de Nantes, Nantes, France. Cyprien, A.L., N. Carcaud, and L. Visset. 2001. Etude paléoenvironnementale du Marais de Distré (Saumurois): géoarchéologie d'une zone himide depuis le Préboréal. Quaternaire 12(1-2):89-101. |
| Marais des Bourbes | 4 | Peat bog | - | Joly, C. 2006. Histoire de la végétation dans l'espace centre-ouest atlantique (France): relations Sociétés Végétation et évolution du trait de côte depuis le Mésolithique récent-final.. Doctoral dissertation. Université de Nantes, Nantes, France. Joly, C. 2004. Histoire végétale d'une tourbière littorale: le Marais des Bourbes (Olonne-sur-Mer, Vendée). [History of vegataion in a coastal peat bog: the Marais des Bourbes (Olonne-sur-Mer, Vendée, France)]. Annales de Paléontologie, 90:187-207. Joly, C., and L. Visset. 2009. Evolution of vegetation landscapes since the Late Mesolithic on the French West Atlantic coast. Review of Palaeobotany and Palynology 154:124-179. |
| Middle North Coombe | 122 | Spring mire | 30 m | Fyfe, R.M., A.G. Brown, and S.J. Rippon. 2004. Characterising the late prehistoric, "Romano-British" and medieval landscape, and dating the emergence of a regionally distinct agricultural system in South West Britain. Journal of Archaeological Science 31(12):1699-1714. |
| Mongan Bog | 40 | Raised bog | 150 ha | Parkes, H.M., and F.J.G. Mitchell. 2000. Vegetation history at Clonmacnoise, Co. Offaly. Biology and Environment: Proceedings of the Royal Irish Academy 100B(1):35-40. |

Table 1 (continued).

| Site name | Elevation  (m a.s.l.) | Site type | Size | Citation |
| --- | --- | --- | --- | --- |
| Montbé | 562 | Bog | - | Jouffroy-Bapicot, I. 2010. Evolution de la végétation du massif du Morvan (Bourgogne - France) depuis la dernière glaciation à partir de l'analyse pollinique. Variations climatiques et impact des activités anthropiques. Doctoral dissertation. Université de Franche-Comté, Besançon, France. Jouffroy-Bapicot, I., B. Vannière, E. Gauthier, H. Richard, F. Monna, and C. Petit. 2013. 7000 years of vegetation history and land-use changes in the Morvan Mountains (France): A regional synthesis. The Holocene 23(12):1888-1902. |
| Morvich |  | Peat basin | - | Davies A (1999) High spatial resolution Holocene vegetation and land-use history in west Glen Affric and Kintail. University of Stirling |
| Moyreen | 120 | Bog | 21 | Plunkett, G. 2009. Land-use patterns and cultural change in the Middle to Late Bronze Age Ireland: inferences from pollen records. Vegetation History Archaeobotany 18:273-295. |
| Nataloup | 515 | Bog | - | Jouffroy-Bapicot, I. 2010. Evolution de la végétation du massif du Morvan (Bourgogne - France) depuis la dernière glaciation à partir de l'analyse pollinique. Variations climatiques et impact des activités anthropiques. Doctoral dissertation. Université de Franche-Comté, Besançon, France. Jouffroy-Bapicot, I., B. Vannière, E. Gauthier, H. Richard, F. Monna, and C. Petit. 2013. 7000 years of vegetation history and land-use changes in the Morvan Mountains (France): A regional synthesis. The Holocene 23(12):1888-1902. |
| Oudon | 17 | Marsh | - | Cyprien, A.L. 2001. Chronologie de l'interaction de l'homme et du milieu dans l'espace central et aval de la Loire (Ouest de la France). Doctoral dissertation. Université de Nantes, Nantes, France. Cyprien, A.L., L. Visset, and N. Carcaud. 2004. Evolution of vegetation landscapes during the Holocene in the central and downstream Loire basin (Western France). Vegetation History and Archaeobotany 13(3):181-196. |
| Owenduff | 150 | Bog | 6000 ha | Plunkett, G. 2009. Land-use patterns and cultural change in the Middle to Late Bronze Age Ireland: inferences from pollen records. Vegetation History Archaeobotany 18:273-295. |
| Paleochenal de Neublans | 184 | Riverine | - | Gauthier, E. 2001. Evolution de l'impact de l'homme sur la végétation du massif jurassien au cours des quatre derniers millénaires. Nouvelles données palynologiques. Doctoral dissertation. Université de Franche-Comté, Besançon, France. |
| Palü Lunga ob Ramosch | 1903 | Spring mire | 10 x 10 m | Welten, M. 1982. Pollenanalytische Untersuchungen zur Vegetationsgeschichte des Schweizerischen Nationalparks. Ergebnisse der wissenschaftlichen Untersuchungen im Schweizerischen Nationalpark 16:1-43. |

Table 1 (continued).

| Site name | Elevation  (m a.s.l.) | Site type | Size | Citation |
| --- | --- | --- | --- | --- |
| Pannel Bridge | 3 | Former fen | - | Waller, M.P. 1987. The Flandrian vegetational history and environmental development of the Brede and Panel Valleys East Sussex. Ph.D. Dissertation. Polytechnic of North London, London, England, United Kingdom.. Doctoral dissertation. Polytechnic of North London, London, United Kingdom. Waller, M.P. 1993. Flandrian vegetational history of south- eastern England. Pollen data from Pannel Bridge, East Sussex. New Phytologist 124(2):345-369. |
| Port des Lamberts | 710 | Bog | - | Jouffroy-Bapicot, I. 2010. Evolution de la végétation du massif du Morvan (Bourgogne - France) depuis la dernière glaciation à partir de l'analyse pollinique. Variations climatiques et impact des activités anthropiques.. Doctoral dissertation. Université de Franche-Comté, Besançon, France. Jouffroy-Bapicot, I., B. Vannière, E. Gauthier, H. Richard, F. Monna, and C. Petit. 2013. 7000 years of vegetation history and land-use changes in the Morvan Mountains (France): A regional synthesis. The Holocene 23(12):1888-1902. Monna, F., C. Petit, J.P. Guillaumet, I. Jouffroy-Bapicot, C. Blanchot, J. Dominik, R. Losno, H. Richard, J. Levèque, and C. Chateau. 2004. History and Environmental Impact of Mining Activity in Celtic Aeduan Territory Recorded in a Peat Bog (Morvan, France). Research 38(3):665-673. |
| Rynholec | 478 | Marsh | - | Pokorný, P. 2005. Role of man in the development of Holocene vegetation in Central Bohemia. Preslia 77(1):113-128. |
| Sägistalsee | 1940 | Lake | 7.3 ha | Van der Knaap, W.O., J.F.N. van Leeuwen, A. Fankhauser, and B. Ammann. 2000. Palynostratigraphy of the last centuries in Switzerland based on 23 lake and mire deposits: chronostratigraphic pollen markers, regional patterns, and local histories. Review of Palaeoecology and Palynology 108(6):85-142. [DOI: 10.1191/0959683605hl852ft] |
| Sheheree Bog | 61 | Bog | 1.96 ha | Mitchell, F.J.G., and T. Cooney. 2004. Vegetation history in the Killarney valley. Pages 481-493 in W. O'Brien, editor. Ross Island: mining metal and society in early Ireland (Bronze age studies, 6). |
| Silberhohl | 180 | Peatland | 150 m | Chen, S.-H. 1988. Neue Untersuchungen über die spät- und postglaziale Vegetationsgeschichte im Gebiet zwischen Harz und Leine (BRD). Flora 181:147-177. |
| Sluggan Moss | 52 | Bog | 600 ha | Smith, A.G., and I.C. Goddard. 1991. A 12500 years record of vegetational history at Sluggan Bog, County Antrim, Northern Ireland (incorporating a pollen zone scheme for the non-specalist). New Phytologist 118:167-187. https://www.jstor.org/stable/2557698. |

Table 1 (continued).

| Site name | Elevation  (m a.s.l.) | Site type | Size | Citation | |
| --- | --- | --- | --- | --- | --- |
| Soppensee | 596 | Lake | 24 ha | Hajdas, I., and A. Michczynski. 2010. Age-depth model of Lake Soppensee (Switzerland) based on the high-resolution C14 chronology compared with the varve chronology. Radiocarbon 52:1027-1040. Lotter, A.F. 1999. Late-glacial and Holocene vegetation history and dynamics as shown by pollen and plant macrofossil analyses in annually laminated sediments from Soppensee, central Switzerland. Vegetation History & Archaeobotany 8:165-184. |  |
| Sources de l'Yonne | 740 | Bog | - | Jouffroy-Bapicot, I. 2010. Evolution de la végétation du massif du Morvan (Bourgogne - France) depuis la dernière glaciation à partir de l'analyse pollinique. Variations climatiques et impact des activités anthropiques.. Doctoral dissertation. Université de Franche-Comté, Besançon, France. Jouffroy-Bapicot, I., B. Vannière, E. Gauthier, H. Richard, F. Monna, and C. Petit. 2013. 7000 years of vegetation history and land-use changes in the Morvan Mountains (France): A regional synthesis. The Holocene 23(12):1888-1902. | |
| Tišice | 160 | Lake | - | Dreslerová, D., E. Břízová, E. Růžičková, and A. Zeman. 2004. Holocene environmental processes and alluvial archaeology in the middle Labe (Elbe) valley. Pages 121-171 in M. Gojda, editor. Ancient landscape, settlement dynamics and non-destructive archaeology. Academia, nakladatelstvi Academie ved ceske republiky, Prague, Czech Republic. Pokorný, P. 2005. Role of man in the development of Holocene vegetation in Central Bohemia. Preslia 77(1):113-128. | |
| Tlstá hora | 460 | Spring fen | - | Rybníčková, E., P. Hájková, and K. Rybníček. 2005. The origin and development of spring fen vegetation and ecosystems - palaeogeobotanical results. Pages 29-57 in A. Poulíčková, M. Hájek, and K. Rybníček, editors. Ecology and palaeoecology of spring fens of the West Carpathians. Palacký University Olomouc, Academy of Sciences of the Czech Republic, Masaryk University Brno, Olomouc. Rybníček, K., and E. Rybníčková. 2008. Upper Holocene dry land vegetation in the Moravian–Slovakian borderland (Czech and Slovak Republics). Vegetation History and Archaeobotany 17(6):701–711. | |
| Torran Beithe |  | Blanket peat | - | Davies A (1999). High spatial resolution Holocene vegetation and land-use history in west Glen Affric and Kintail. University of Stirling | |

Table 1 (continued).

| Site name | Elevation  (m a.s.l.) | Site type | Size | Citation |
| --- | --- | --- | --- | --- |
| Vertonne | 3 | Marsh | - | Joly, C. 2006. Histoire de la végétation dans l'espace centre-ouest atlantique (France): relations Sociétés/Végétation et évolution du trait de côte depuis le Mésolithique récent-final. Doctoral dissertation. Université de Nantes, Nantes, France. Joly, C., and L. Visset. 2009. Evolution of vegetation landscapes since the Late Mesolithic on the French West Atlantic coast. Review of Palaeobotany and Palynology 154:124-179. |
| Vladař | 612 | Reservoir | - | Pokorný, P., N. Boenke, M. Chytráček, K. Nováková, J. Sádlo, J. Veselý, P. Kuneš, and V. Jankovská. 2006. Insight into the environment of a pre-Roman Iron Age hillfort at Vladař, Czech Republic, using a multi-proxy approach. Vegetation History and Archaeobotany 15(4):419-433. [DOI: 10.1007/s00334-006-0064-8] |
| Vrbka | 180 | Peatland | - | Pokorný, P. 2016. Contributions to the European Pollen Database 29. Vrbka (Czech Republic): pollen record of secondary steppe vegetation development within the Bronze Age agricultural landscape. Grana 55(3):246-249. [DOI: 10.1080/00173134.2015.1120342] |
| Windmill Rough | 263 | Spring mire | - | Fyfe, R.M., A.G. Brown, and S.J. Rippon. 2004. Characterising the late prehistoric, "Romano-British" and medieval landscape, and dating the emergence of a regionally distinct agricultural system in South West Britain. Journal of Archaeological Science 31(12):1699-1714. |
| Zahájí | 232 | Spring mire | - | Pokorný, P. 2005. Role of man in the development of Holocene vegetation in Central Bohemia. Preslia 77(1):113-128. |

# Appendix S3: Characteristics of the pollen taxa

Table 2 Overview of the available information per pollen taxon and occurrence in pollen records. PPE = pollen productivity estimate from Wieczorek and Herzschuh (2020).

| **Pollen taxon** | **PPE** | **Occurrence in pollen records** | **Number of trait observations** | **Number of species in pollen taxon** |
| --- | --- | --- | --- | --- |
| Abies | 6.88 | 66.67 | 703 | 32 |
| Acer | 0.23 | 75.64 | 1704 | 24 |
| Alnus | 8.46 | 100 | 309 | 14 |
| Apiaceae | 2.13 | 96.15 | 3129 | 164 |
| Artemisia-type | 11.67 | 96.15 | 342 | 40 |
| Asteraceae | 1.42 | 100 | 9338 | 644 |
| Betula | 7.2 | 100 | 2669 | 17 |
| Brassicaceae | 0.48 | 96.15 | 3332 | 211 |
| Campanulaceae | 2.29 | 75.64 | 809 | 92 |
| Carpinus | 4.31 | 78.21 | 192 | 9 |
| Caryophyllaceae | 21.74 | 98.72 | 3680 | 186 |
| Castanea | 5.87 | 43.59 | 333 | 5 |
| Cerealia | 3.51 | 62.82 | 341 | 18 |
| Convolvulaceae | 0.18 | 30.77 | 328 | 40 |
| Cornaceae | 1.72 | 35.9 | 386 | 13 |
| Corylus-type | 1.78 | 100 | 446 | 8 |
| Cyperaceae | 1.82 | 100 | 3939 | 251 |
| Ericales | 0.83 | 98.72 | 3063 | 84 |
| Fabaceae | 0.3 | 93.59 | 6991 | 218 |
| Fagus | 2.92 | 100 | 1120 | 3 |
| Fraxinus | 2.42 | 97.44 | 719 | 8 |
| Humulus | 16.43 | 74.36 | 30 | 1 |
| Juglans | 3.28 | 73.08 | 292 | 7 |
| Juniperus-type | 14.3 | 67.95 | 245 | 15 |
| Lamiaceae | 1.06 | 89.74 | 3398 | 181 |
| Larix | 3.44 | 17.95 | 918 | 8 |
| Liliaceae-type | 1.49 | 76.92 | 164 | 61 |
| Moraceae | 1.1 | 1.28 | 165 | 11 |
| Orobanchaceae | 0.33 | 71.79 | 1084 | 135 |
| Picea | 5.96 | 66.67 | 2190 | 27 |
| Pinus | 14.64 | 98.72 | 7097 | 36 |
| Plantaginaceae | 3.54 | 100 | 2828 | 127 |
| Poaceae | 1 | 100 | 11369 | 380 |
| Populus | 1.59 | 56.41 | 658 | 18 |
| Quercus | 3.58 | 100 | 9077 | 35 |
| Ranunculaceae | 2.4 | 100 | 1939 | 137 |
| Rosaceae | 0.88 | 98.72 | 6255 | 388 |
| Rubiaceae | 1.67 | 85.9 | 1834 | 68 |
| Rumex | 2.01 | 100 | 1661 | 49 |
| Salix | 1.3 | 98.72 | 2170 | 106 |
| Sambucus nigra | 1.3 | 28.21 | 166 | 1 |

Table 2 (continued)

| **Pollen taxon** | **PPE** | **Occurrence in pollen records** | **Number of trait observations** | **Number of species in pollen taxon** |
| --- | --- | --- | --- | --- |
| Sanguisorba-type | 24.07 | 53.85 | 248 | 4 |
| Thalictrum | 4.65 | 79.49 | 185 | 14 |
| Thymelaceae | 33.05 | 12.82 | 114 | 20 |
| Tilia | 1.02 | 94.87 | 661 | 16 |
| Ulmus | 2.24 | 100 | 401 | 9 |
| Urtica | 10.52 | 93.59 | 362 | 9 |

# Appendix S4: JAGS code

JAGS code for CWM calculation.

# Data

# Nyr = number of observations in the pollen data

# Ntax = number of taxa in the pollen data

# Nvar = number of traits in the trait data

# N = number of trait observations, thus the number of rows of zTrait

# Ab = abundance matrix with observations as rows and taxa as species, in this case a matrix of Nyr rows and Ntax columns

# zTrait = matrix of standardized trait observations of Nvar columns

# Tax = factor of Ntax levels with same length as N

# sdOrig = vector of length Nvar with the standard deviations of the trait data, for converting to the original scale

# meanOrig = vector of length Nvar with the mean of the trait data, for converting to the original scale

# zRscal = parameter of the Wisharts distributrion (dwish). zRscal is a vector of Ntax. zRscal is specified as degrees of freedom + 1 so to give a flat prior on the correlation parameters

# zRmat = parameter of the Wisharts distributrion (dwish).

# zRmat = a list of covariance matrices of length Ntax. Every matrix is of size Nvar by Nvar.

model {

## Pollen taxonomic level

# Likelihood

for (i in 1:N) {

zTrait[i, 1:Nvar] ~ dmnorm(zMu[Tax[i], 1:Nvar], zInvCovMat[1:Nvar, 1:Nvar, Tax[i]])

}

# Priors

for(taxID in 1:Ntax){

for(varID in 1:Nvar){

zMu[taxID, varID] ~ dnorm(0, 10^-4)

}

zInvCovMat[1:Nvar, 1:Nvar, taxID] ~ dwish(zRmat[1:Nvar, 1:Nvar, taxID], zRscal[taxID])

}

# Convert taxon mean and sd to original scale:

# Convert invCovMat to sd

for(taxID in 1:Ntax){

zCovMat[1:Nvar, 1:Nvar, taxID] <- inverse(zInvCovMat[1:Nvar, 1:Nvar, taxID])

for (varID in 1:Nvar ) {

zSigma[taxID, varID] <- sqrt(zCovMat[varID, varID, taxID])

}

}

for (taxID in 1:Ntax){

for (varID in 1:Nvar) {

sigma[taxID, varID] <- zSigma[taxID,varID] * sdOrig[varID]

mu[taxID, varID] <- zMu[taxID, varID] * sdOrig[varID] + meanOrig[varID]

}

}

## Community level

for (i in 1:Nyr){

zCWM[i,1:Nvar] ~ dmnorm(zMu[r[i],1:Nvar], zInvCovMat[1:Nvar, 1:Nvar, r[i]])

r[i] ~ dcat(Ab[i, 1:Ntax])

}

# Convert cwm to original scale

for (i in 1:Nyr){

for (varID in 1:Nvar) {

CWM[i,varID] <- zCWM[i,varID] * sdOrig[varID] +

meanOrig[varID]

}

}

JAGS code for the general additive model:

$${CWT}_{s,y}^{mean} \sim Normal(\beta_{0}{+ f(time)+\alpha_{s},CWT}_{s,y}^{sd})$$

This model was partly generated by the *jagam* function from the *mgcv* R package (Wood 2017).

model {

# data

# y = mean of CWT (calculated previously)

# sd = standard deviation of CWT (calculated previously)

# n = number of observations, e.g. length of y and sd

# site = factor of nsite levels and length n

# nsite = number of sites

mu0 <- X %*% b ## expected response

for (i in 1:n) { y[i] ~ dnorm(mu[i], tau[i])

# parameterize tau

tau[i] <- 1/sd[i]^2

}

# random effect of site

for (i in 1:n) {

mu[i] <- mu0[i] + re[site[i]]

}

for(i in 1:nsite){ re[i] ~ dnorm(0, tau.re)}

tau.re ~ dgamma(0.001, 0.001)

# Smooths

for (i in 1:1) { b[i] ~ dnorm(0, 10^-4) }

# prior for s(Time.BP)

for (i in 2:9) { b[i] ~ dnorm(0, lambda[1]) }

for (i in 10:10) { b[i] ~ dnorm(0, lambda[2]) }

# smoothing parameter priors

for (i in 1:2) {

lambda[i] ~ dgamma(.001,.001)

rho[i] <- log(lambda[i])

}

}

JAGS code for the general additive model:

$${CMW}_{s,y}^{mean} \sim Normal\left( \beta_{0}{+ f\left( agriculture \right)+f\left( temperature \right)+\alpha_{s}, CMW}_{s,y}^{sd} \right)$$

This model was partly generated by the *jagam* function from the *mgcv* R package (Wood 2017).

model {

# data

# y = mean of CWM (calculated previously)

# sd = standard deviation of CWM (calculated previously)

# n = number of observations, e.g. length of y and sd

# site = factor of nsite levels and length n

# nsite = number of sites

mu0 <- X %*% b ## expected response

for (i in 1:n) { y[i] ~ dnorm(mu[i], tau[i])

# parameterize tau

tau[i] <- 1/sd[i]^2

}

# random effect of site

for (i in 1:n) {

mu[i] <- mu0[i] + re[site[i]]

}

for(i in 1:nsite){ re[i] ~ dnorm(0, tau.re)}

tau.re ~ dgamma(0.001, 0.001)

# Smooths

for (i in 1:1) { b[i] ~ dnorm(0,10^-4) }

## prior for s(years.since)

for (i in 2:9) { b[i] ~ dnorm(0, lambda[1]) }

for (i in 10:10) { b[i] ~ dnorm(0, lambda[2]) }

## prior for s(Temperature)

for (i in 11:18) { b[i] ~ dnorm(0, lambda[3]) }

for (i in 19:19) { b[i] ~ dnorm(0, lambda[4]) }

## smoothing parameter priors

for (i in 1:4) {

lambda[i] ~ dgamma(.001,.001)

rho[i] <- log(lambda[i])

}

}

# Appendix S5: Comparison between univariate and multivariate likelihood for CWM calculation


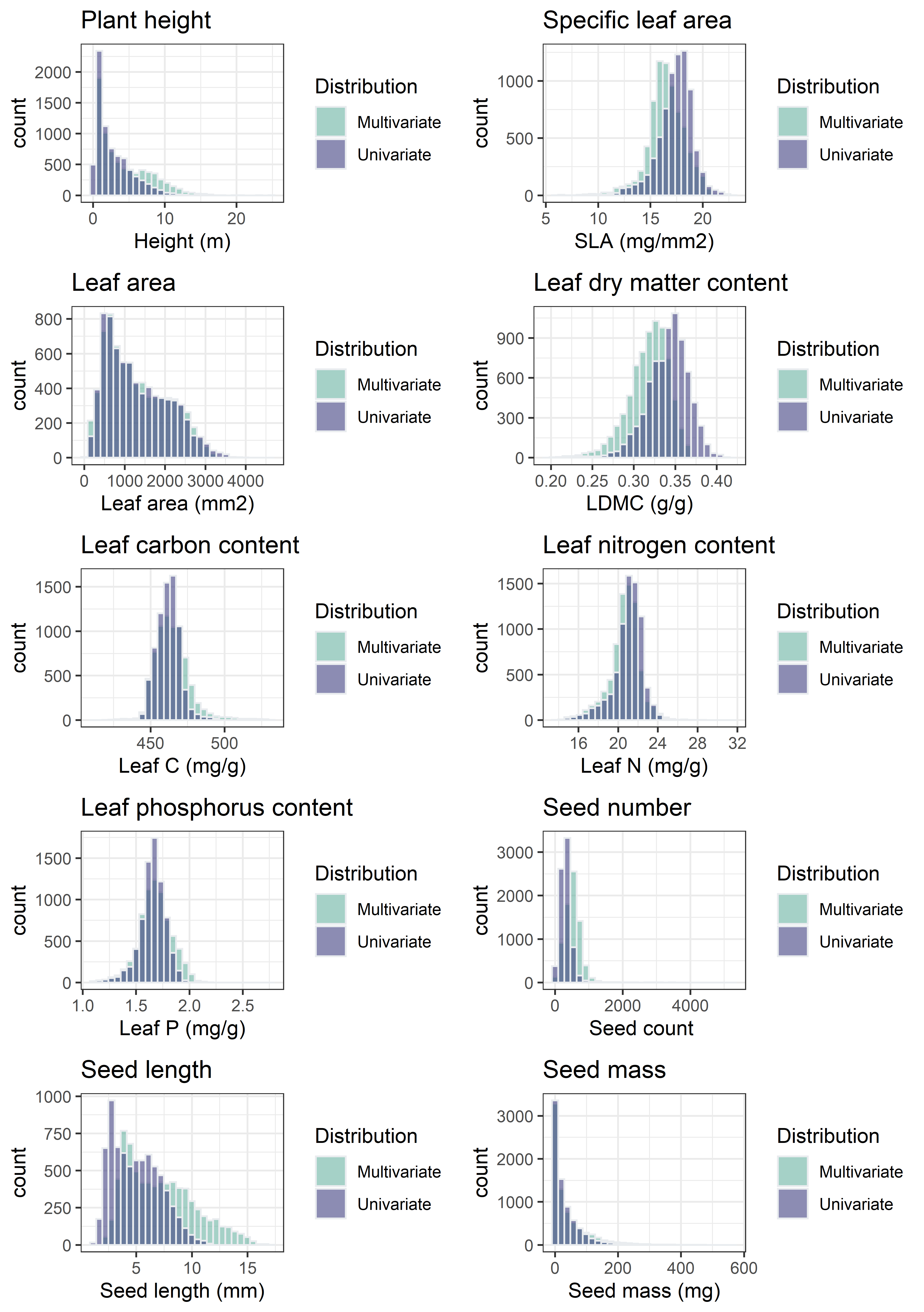


Figure 2 Histograms of reconstructed CWM^mean^ trait values using a univariate or a multivariate distribution for the likelihood.


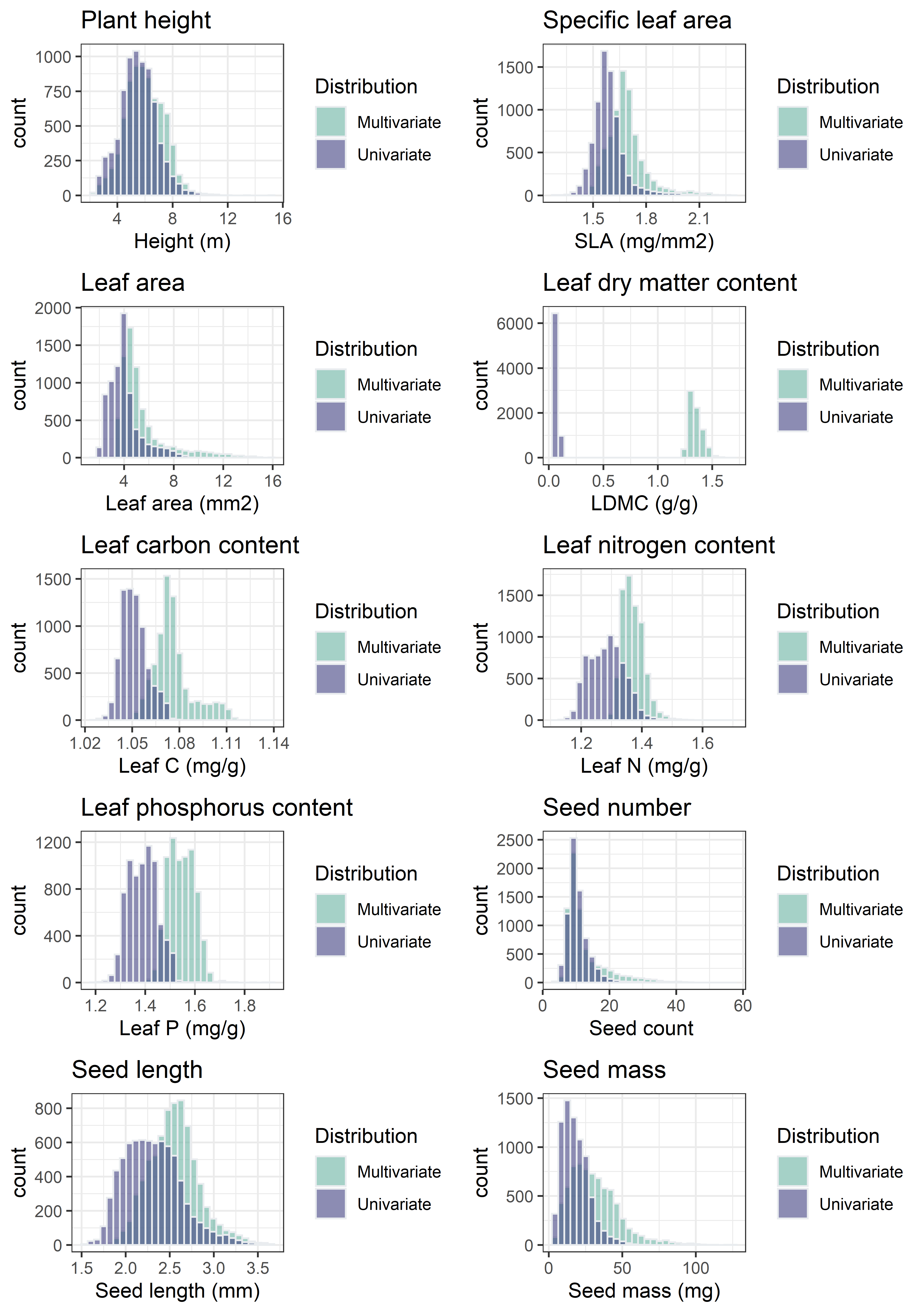


Figure 3 Histograms of reconstructed CWM^sd^ trait values using a univariate or a multivariate distribution for the likelihood.

# Appendix S6: PCA plots


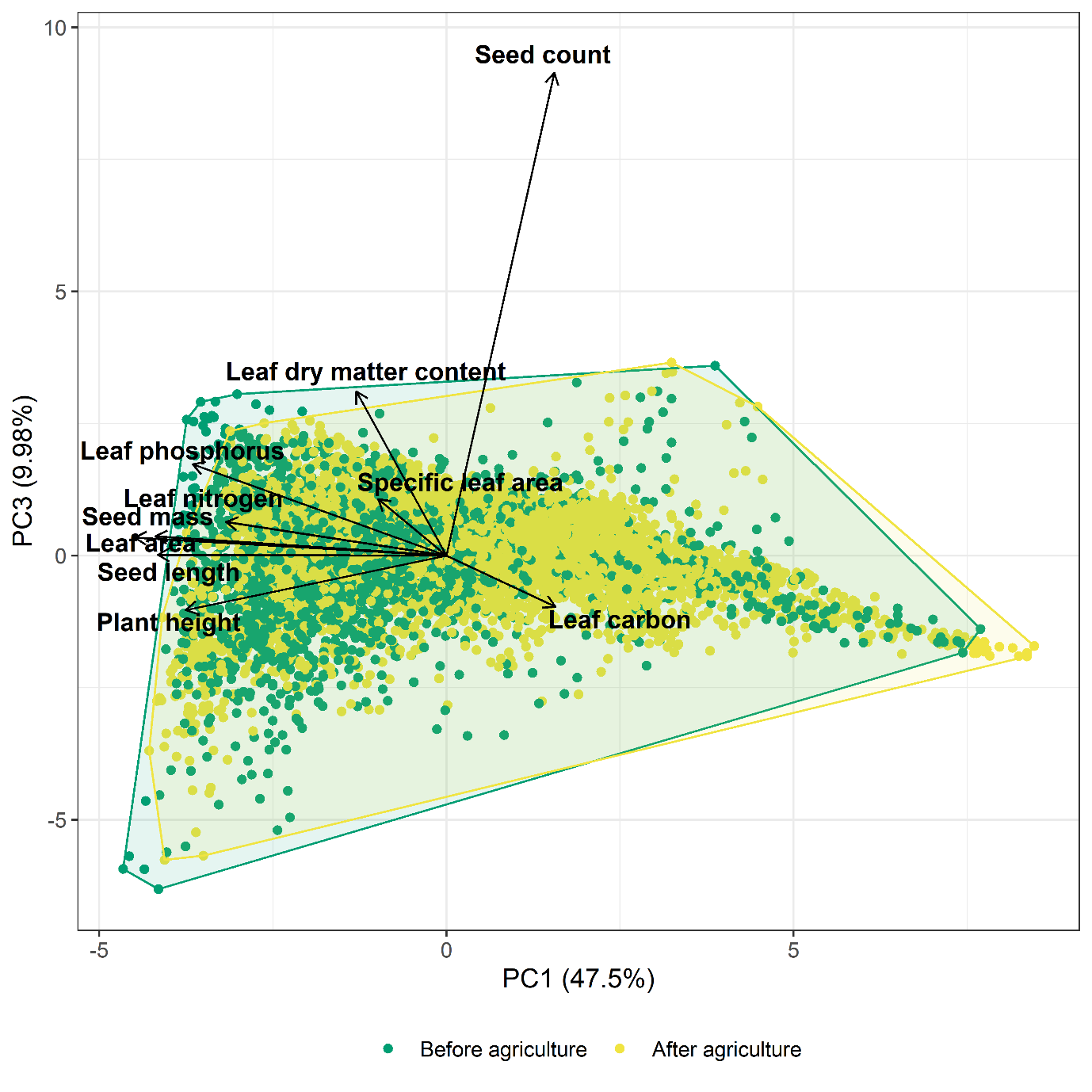


Figure 4 Plot of principal component analysis of community level trait values for principle components 1 and 3. Every colored point represents the reconstructed community trait value. The color indicates whether the observation is from before or after agriculture.


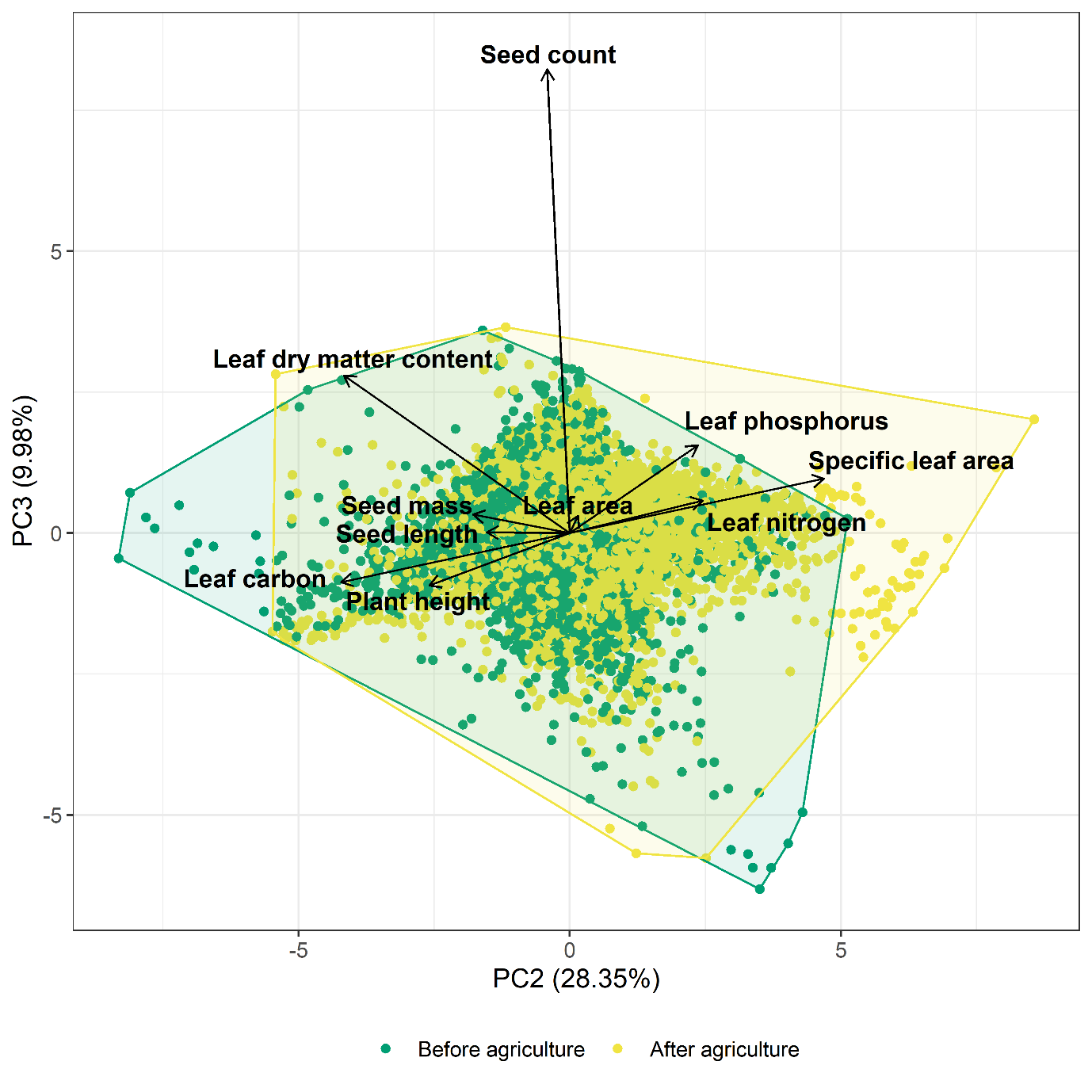


Figure 5 Plot of principal component analysis of community level trait for principle components 2 and 3. Every colored point represents the reconstructed community trait value. The color indicates whether the observation is from before or after agriculture.

To test the sensitivity of the analysis to the inclusion of plant height as a functional trait we performed the PCA on CWM calculation that excluded plant height.


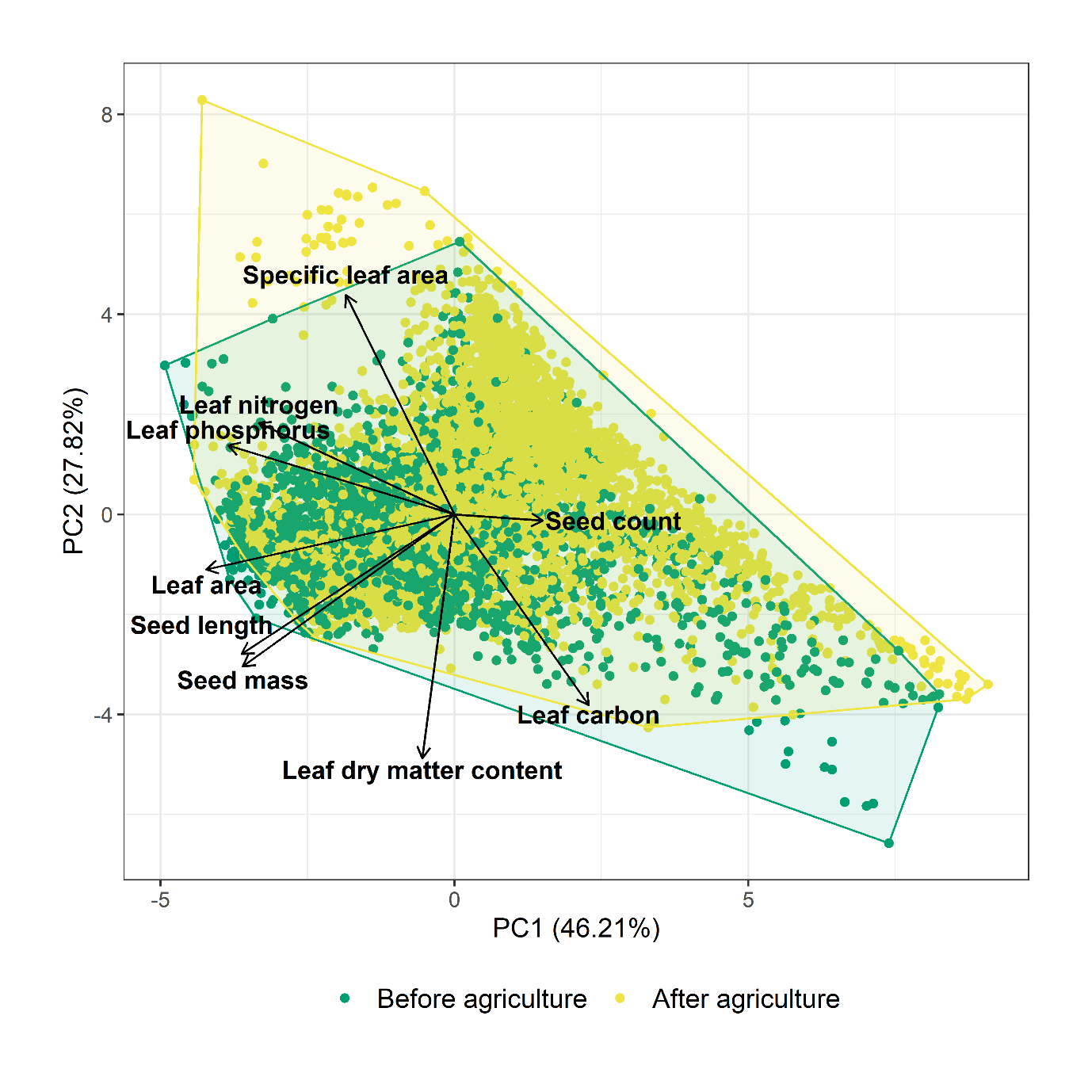


Figure 6 Principal component analysis of community level trait values without plant height, principle components 1 and 2. Every colored point represents the reconstructed community trait value. The color indicates whether the observation is from before or after agriculture.

Table 3 Loadings of the traits on the principle components for the PCA without plant height.

| **Trait** | **PC1** | **PC2** | **PC3** |
| --- | --- | --- | --- |
| **Leaf area** | -0.469 | -0.122 | 0.011 |
| **Leaf phosphorus** | -0.428 | 0.153 | 0.167 |
| **Seed length** | -0.402 | -0.31 | -0.043 |
| **Seed mass** | -0.4 | -0.338 | -0.004 |
| **Leaf nitrogen** | -0.37 | 0.203 | 0.096 |
| **Specific leaf area** | -0.205 | 0.487 | 0.118 |
| **Leaf dry matter content** | -0.061 | -0.542 | 0.259 |
| **Seed count** | 0.167 | -0.014 | 0.934 |
| **Leaf carbon** | 0.253 | -0.423 | -0.089 |
| **Proportion of variance explained (%)** | 46.2 | 27.8 | 10.9 |

# Appendix S7: Evaluation of GAMs

To examine the variability of the smooths, 50 smooth curves from the posterior were drawn (Wood 2016). Figure 7 shows the results for the first GAM for trait change over time.
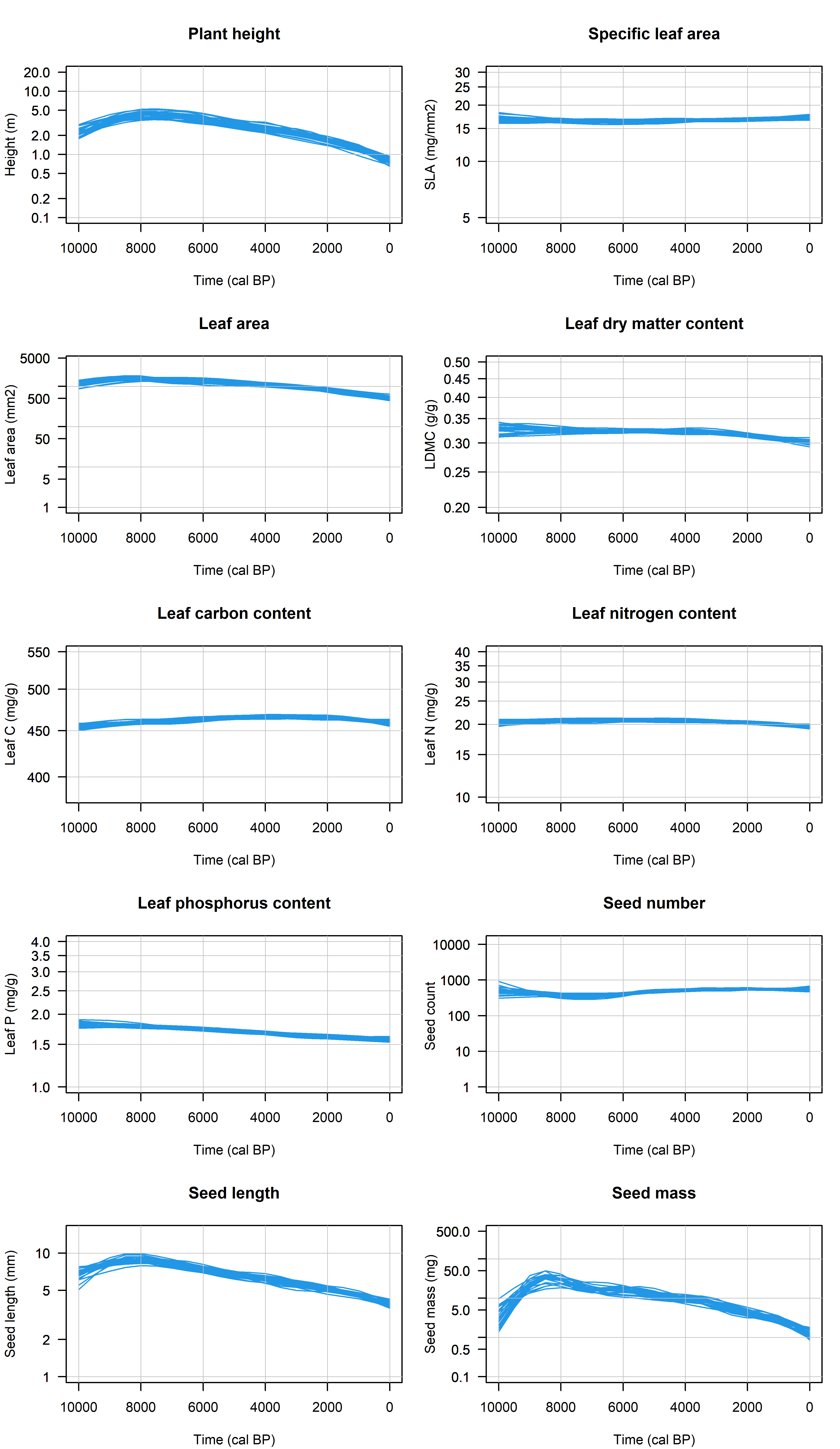


Figure 7 50 random draws from the posterior distributions of the GAM for trait change over time. Every line is a draw from the posterior.

To examine the sensitivity of the GAMs to the choice of sites, we performed reruns of the GAM whilst leaving 1 site at the time. Figure 8 shows the results of this model validation for the first GAM for trait change over time.


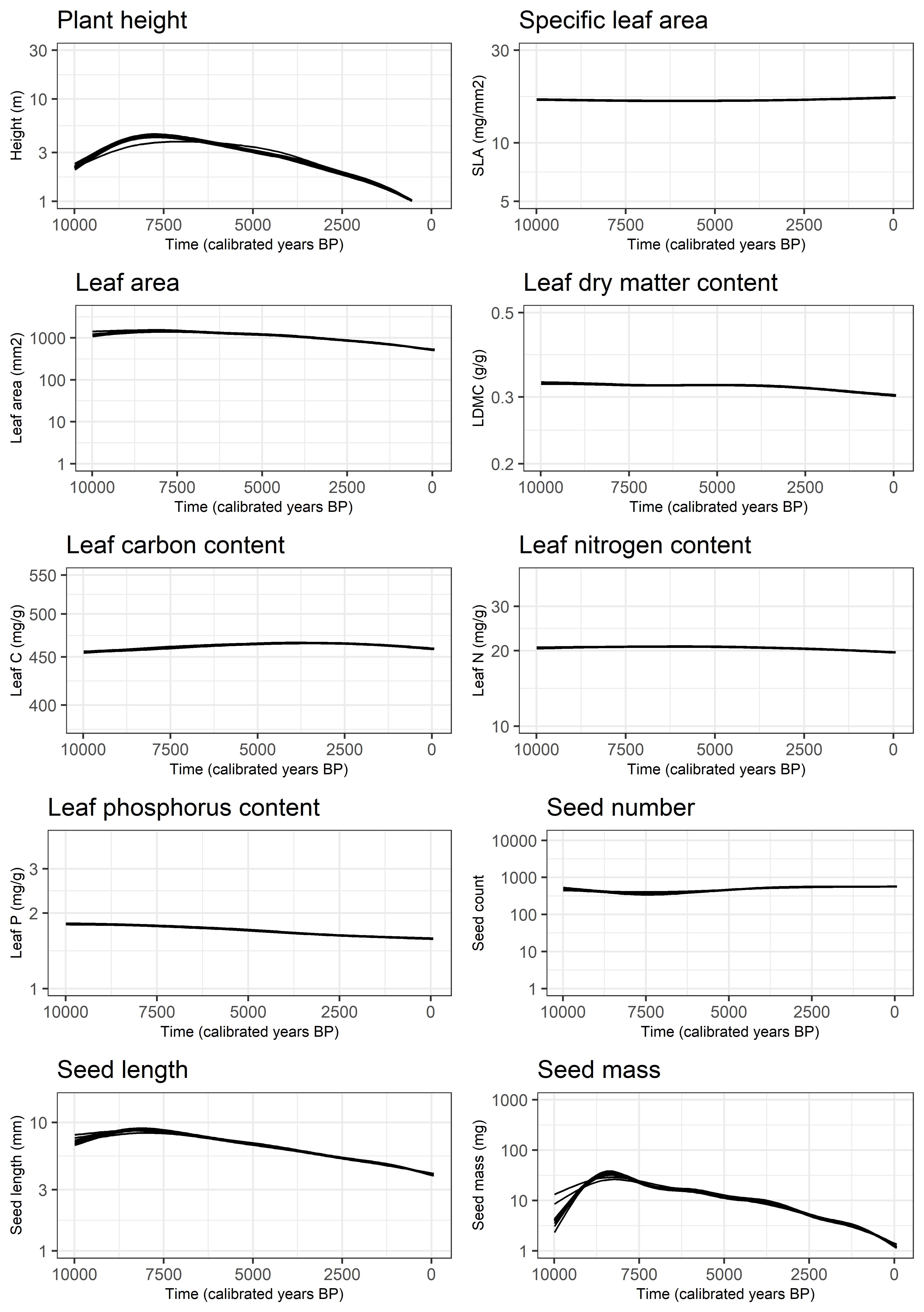


Figure 8 Reruns of GAM for trait change over time for testing the effect of site choice on the model. Every line is a run of the GAM while leaving one site from the dataset out.

To examine the sensitivity of the GAMs to uncertainty in the radiocarbon dating, we performed reruns of the GAM with 50 random draws from the *Bchron* age model. Figure 9 shows the results of this model validation for the first GAM for trait change over time.


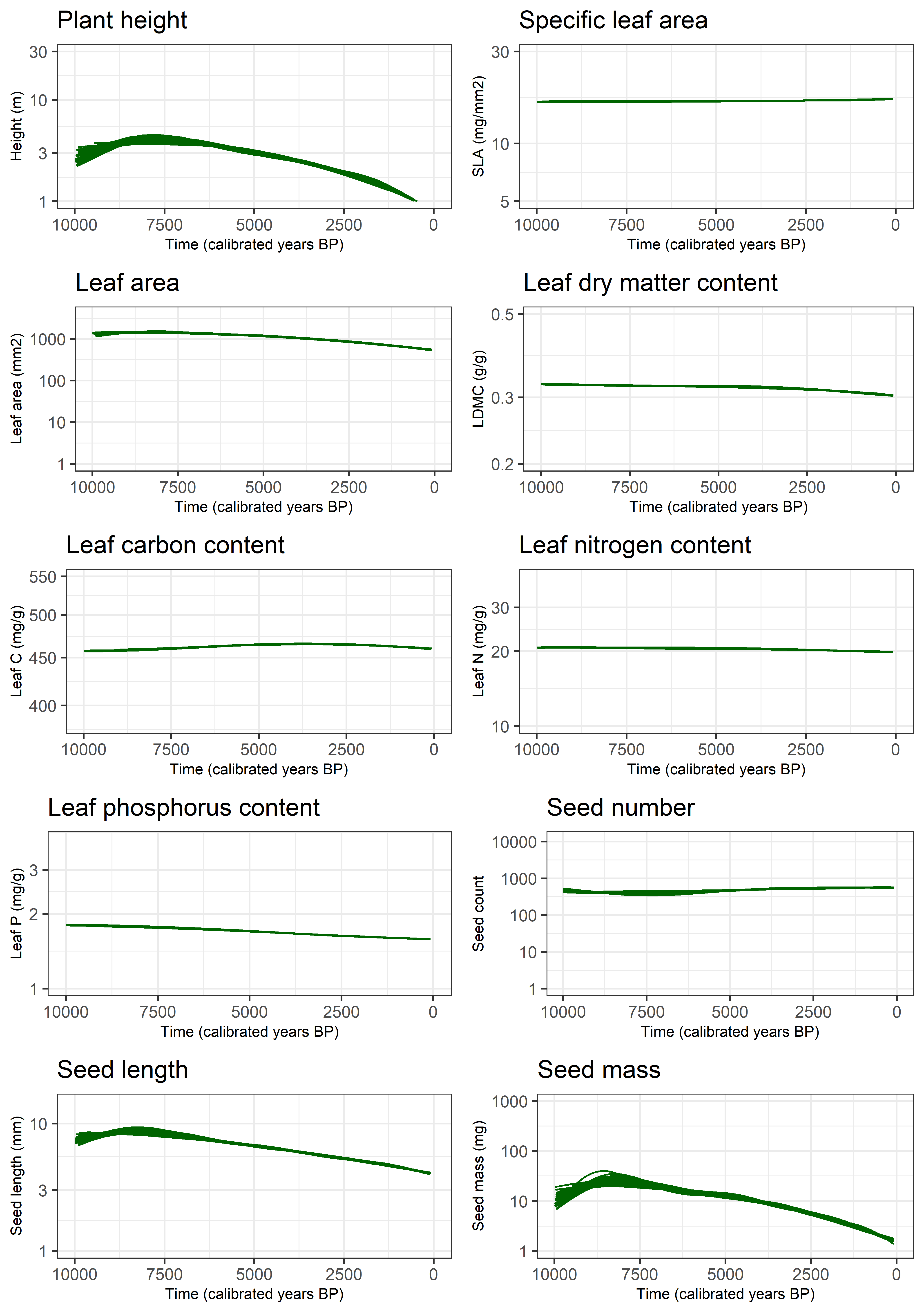


Figure 9 50 reruns of the GAM for trait change over time for testing the effect of age uncertainties on the model. Every line is a GAM run on a random draw from the posterior of the Bchron age models.

To examine the variability of the smooths, 50 smooth curves from the posterior were drawn (Wood 2016). Figure 10 and 11 show the results for the second GAM for trait change since the arrival of agriculture and temperature.


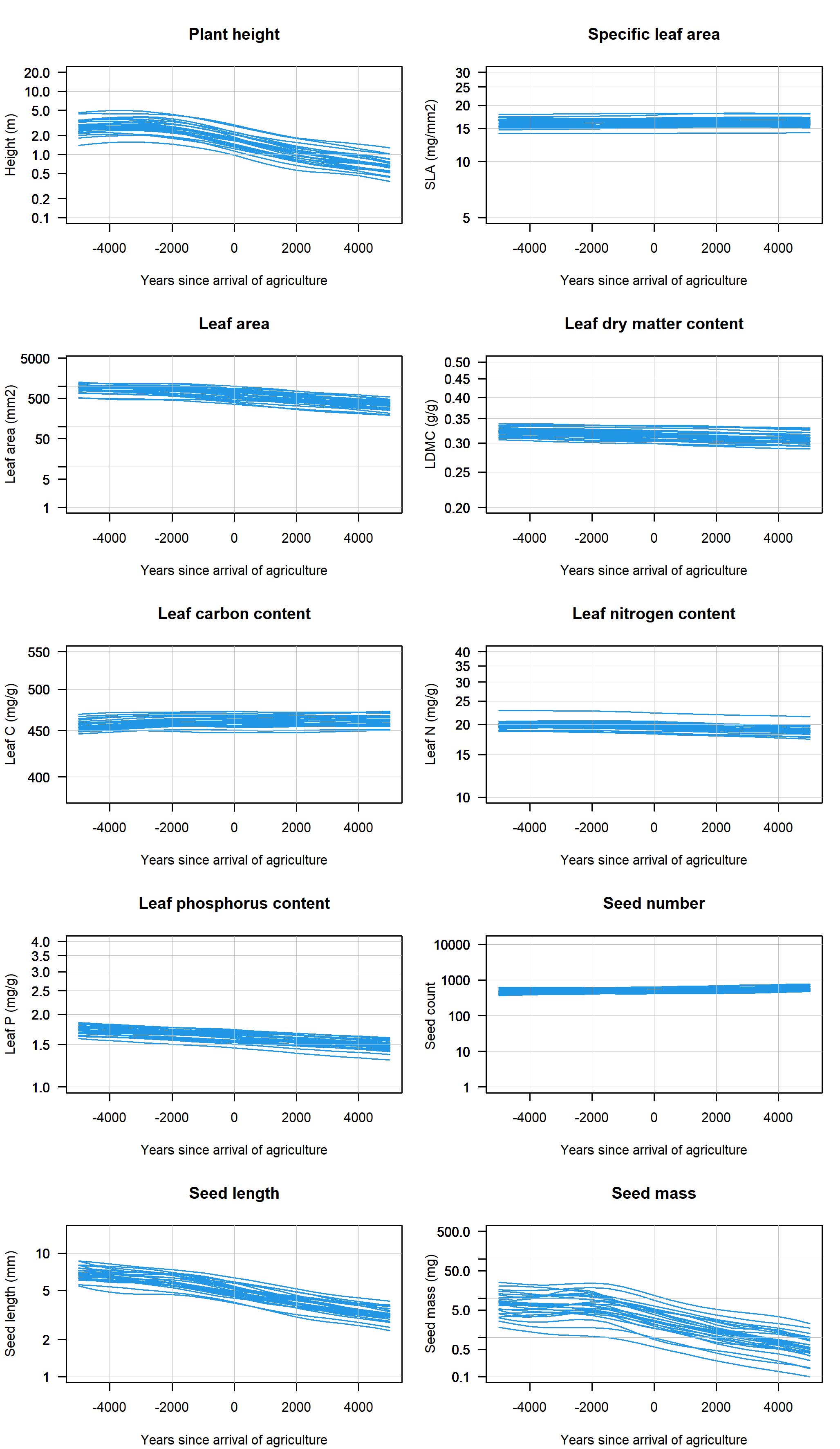


Figure 10 50 random draws from the posterior distributions of the GAM for trait change after the arrival of agriculture and temperature. Partial component plot for years since the arrival of agriculture. Every line is a draw from the posterior.


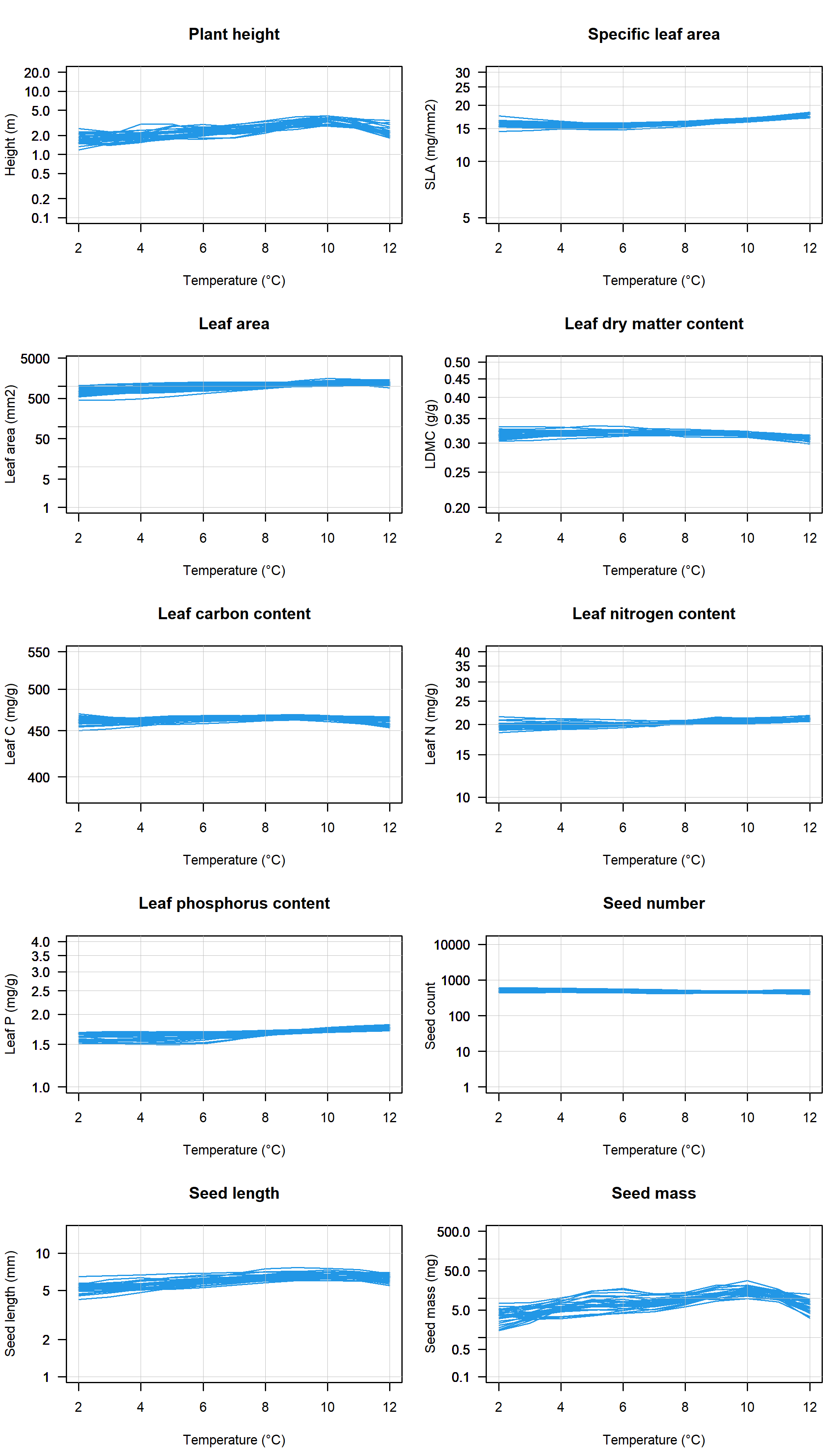


Figure 11 50 random draws from the posterior distributions of the GAM for trait change after the arrival of agriculture and temperature. Partial component plot for temperature. Every line is a draw from the posterior.

To examine the sensitivity of the GAMs to the choice of sites, we performed reruns of the GAM whilst leaving 1 site at the time. Figure 12 and 13 show the results of this model validation for the second GAM for trait change since the arrival of agriculture and temperature.


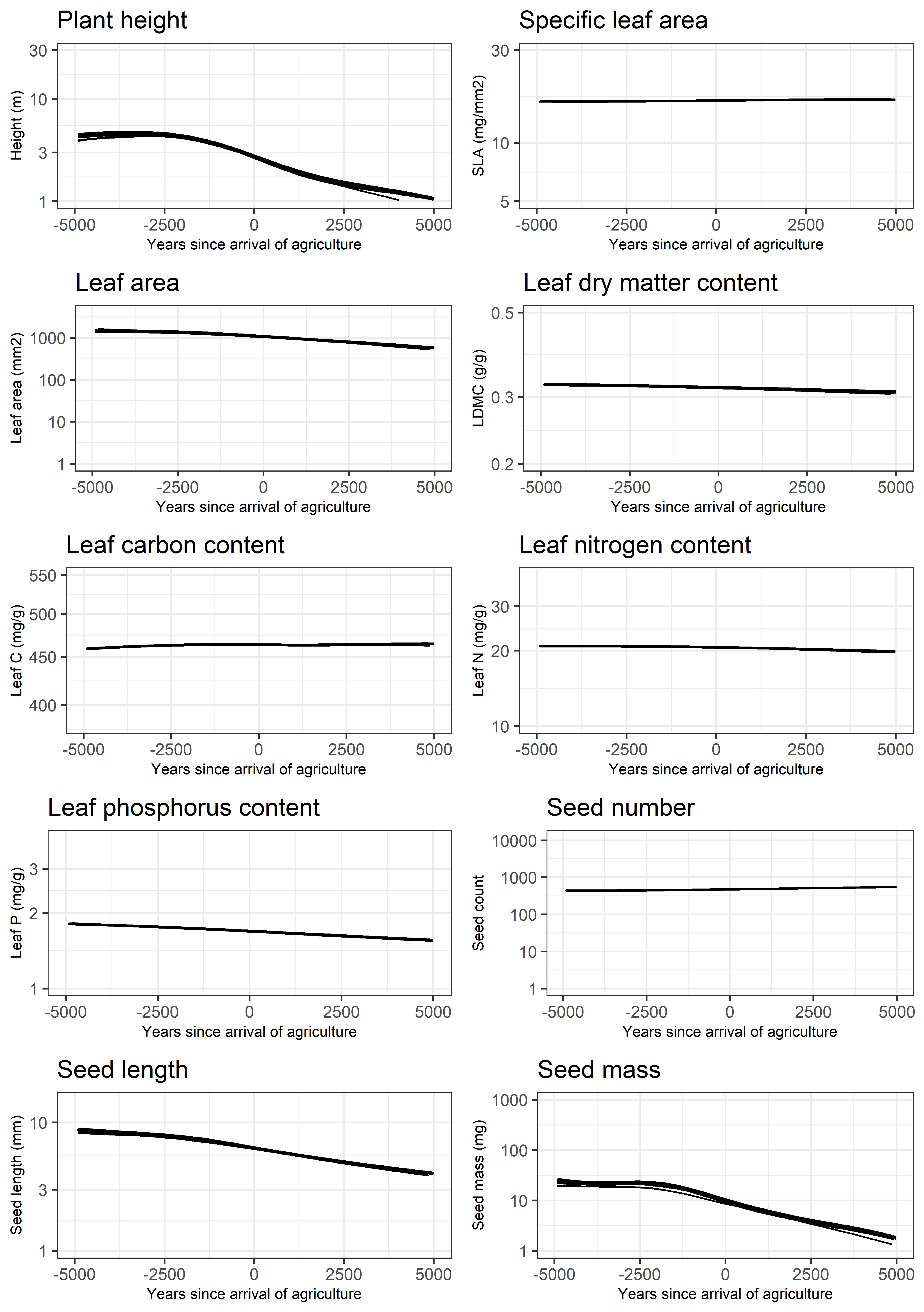


Figure 12 Reruns of GAM for agriculture and temperature for testing the effect of site choice on the model. Partial component plot for years since the arrival of agriculture. Every line is a run of the GAM while leaving one site from the dataset out.


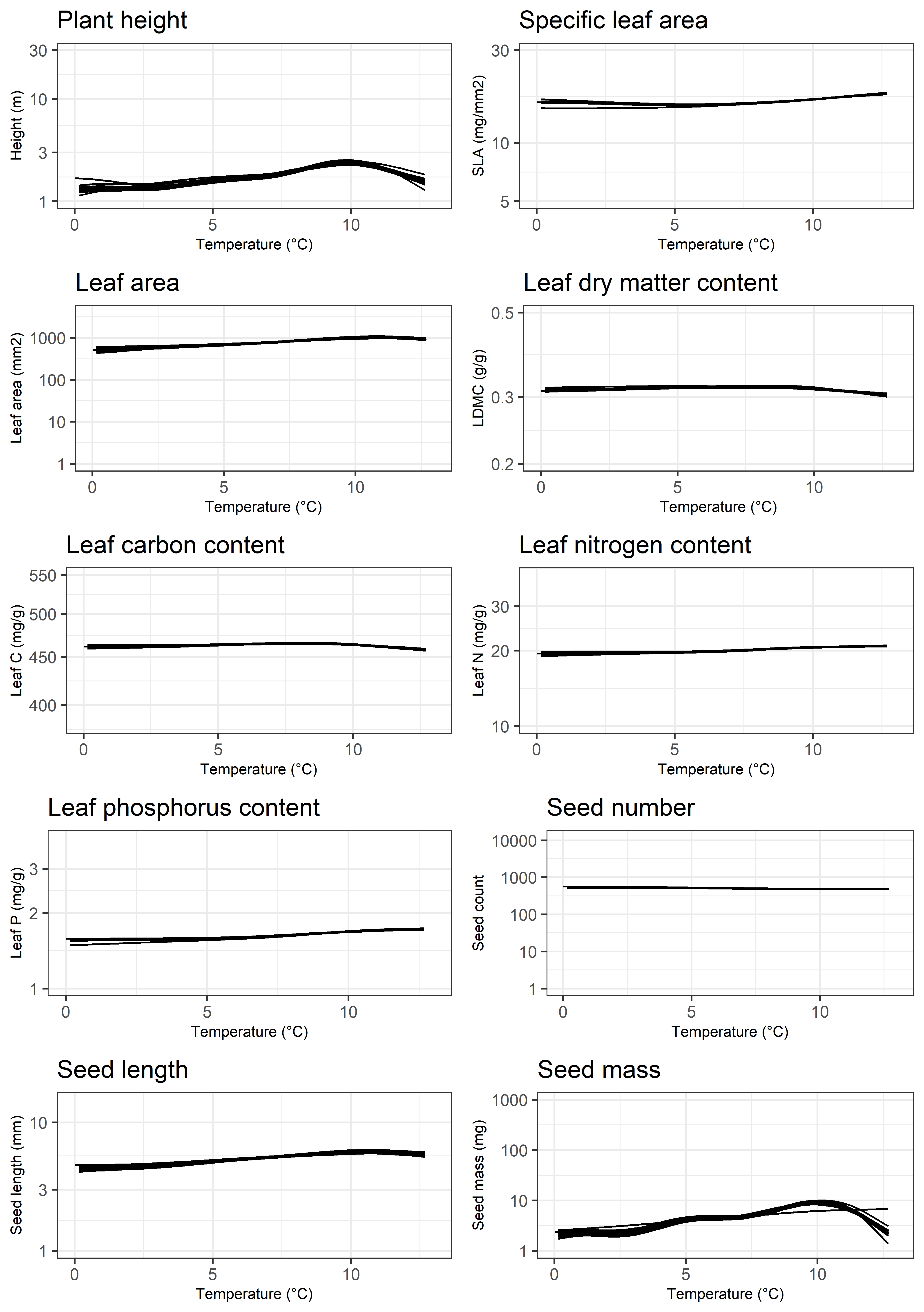


Figure 13 Reruns of GAM for agriculture and temperature for testing the effect of site choice on the model. Partial component plot for temperature. Every line is a run of the GAM while leaving one site from the dataset out.

To examine the sensitivity of the GAMs to uncertainty in the radiocarbon dating, we performed reruns of the GAM with 50 random draws from the *Bchron* age model. Figure 14 and 15 show the results of this model validation for the second GAM for trait change since the arrival of agriculture and temperature.


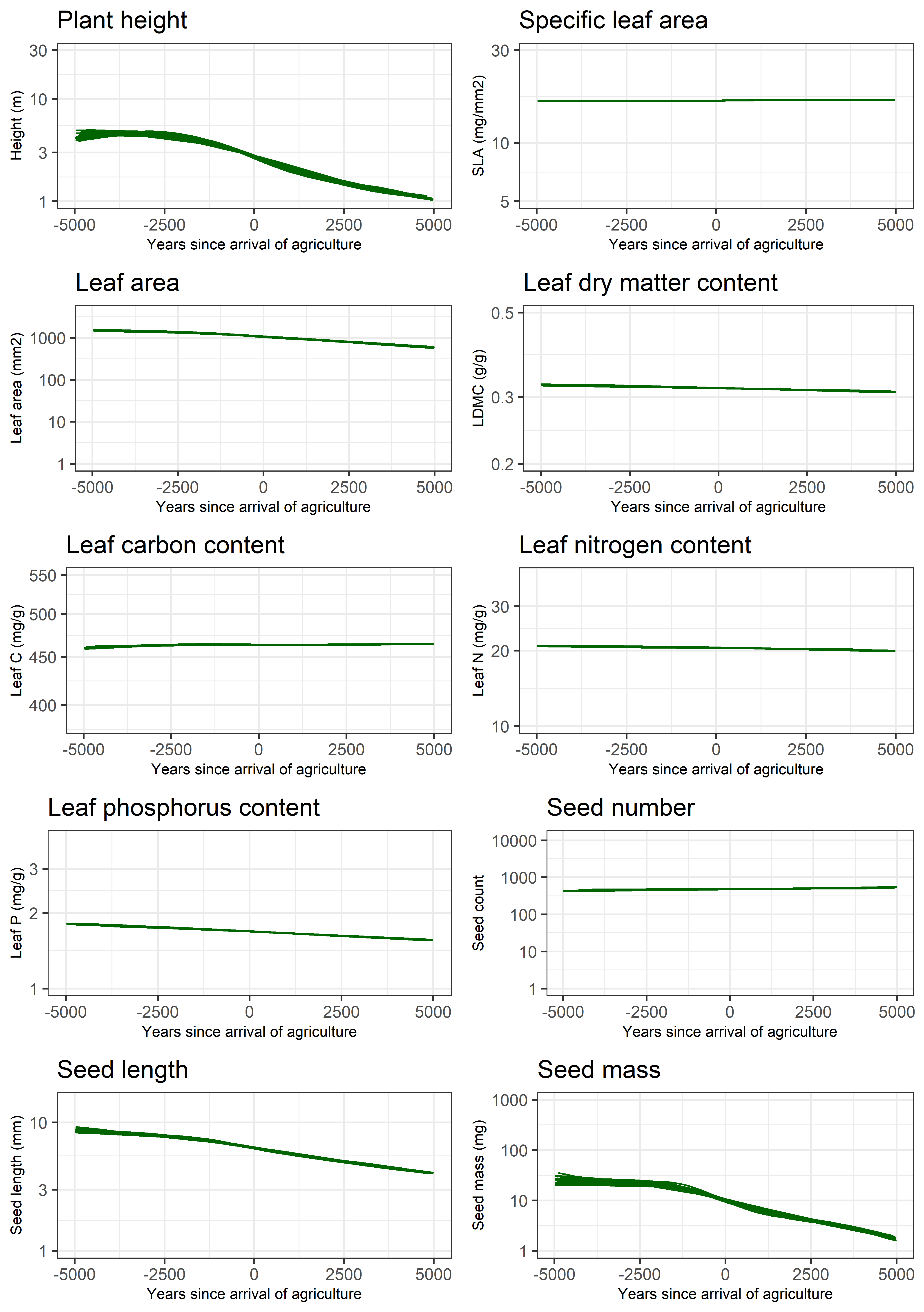


Figure 14 50 reruns of the GAM for trait change over time for testing the effect of age uncertainties on the model. Partial component plot for years since arrival of agriculture. Every line is a GAM run on a random draw from the posterior of the Bchron age models.


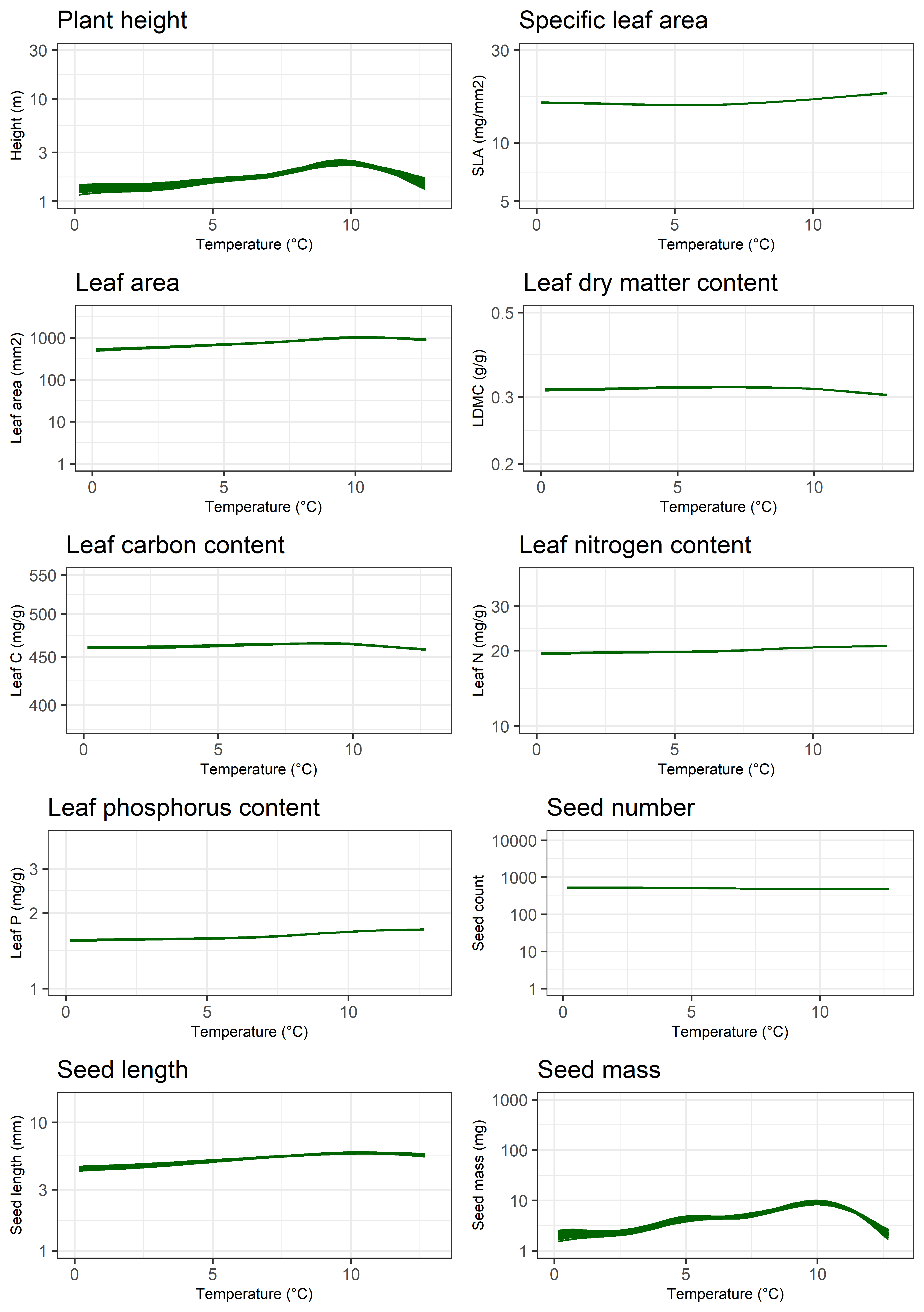


Figure 15 50 reruns of the GAM for trait change over time for testing the effect of age uncertainties on the model. Partial component plot for temperature. Every line is a GAM run on a random draw from the posterior of the Bchron age models

# Appendix S8: Temperature data


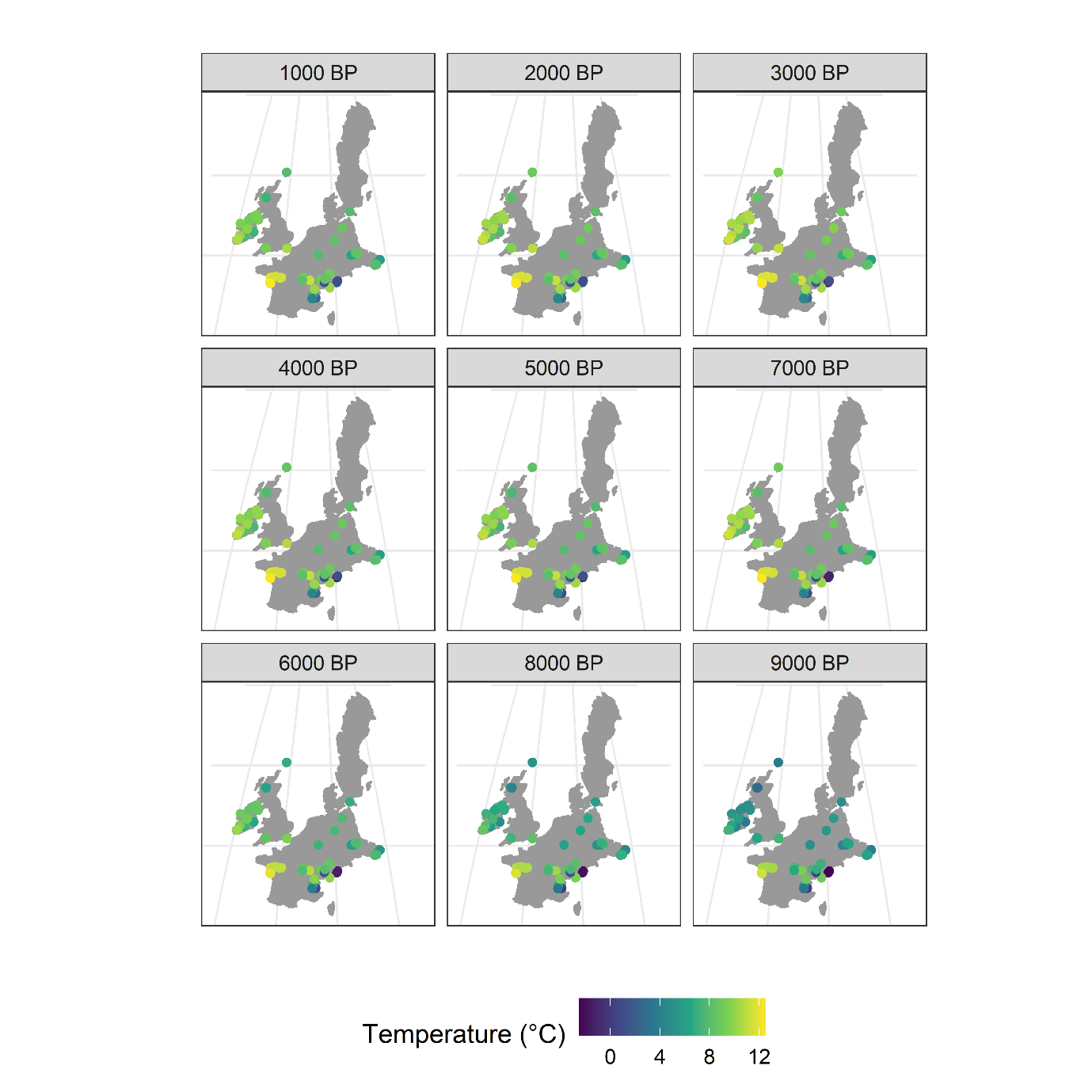


Figure 16 Average mean annual temperature at the pollen sites from the CHELSA-Trace21k dataset.

# Appendix S9: Correlogram CWM trait values


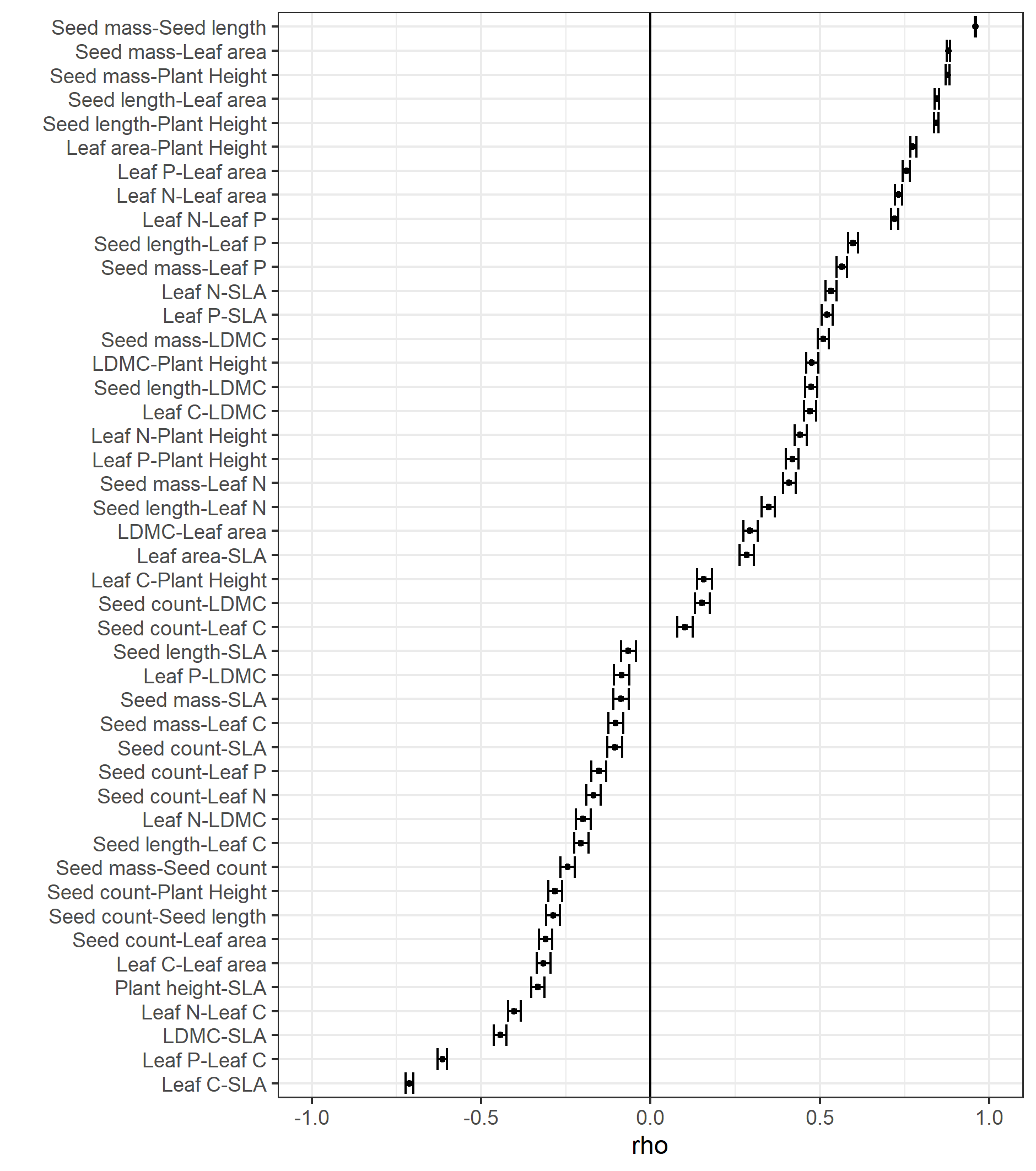


Figure 17 Correlogram of CWM trait values.

# Appendix S10: Taxon level trait estimates

Table 3 Trait estimates on the pollen taxonomic level.

| **Pollen taxon** | **SLA** | **Plant height** | **LA** | **LDMC** | **Leaf carbon** | **Leaf phosphorus** | **Leaf nitrogen** | **Seed length** | **Seed count** | **Seed mass** |
| --- | --- | --- | --- | --- | --- | --- | --- | --- | --- | --- |
|  | mg/mm^2^ | m | mm^2^ | g/g | mg/g | mg/g | mg/g | mm |  | mg |
| *Abies* | 5.07 ± 1.33 | 33.24 ± 2.33 | 31± 2.11 | 0.3 ± 1.17 | 501.81 ± 1.04 | 1.43 ± 1.34 | 13.14 ± 1.3 | 9.25 ± 1.49 | 4936 ± 2.73 | 19.25 ± 4.03 |
| *Acer* | 17.66 ± 1.38 | 16.88 ± 2.27 | 4143± 2.21 | 0.35 ± 1.15 | 465.19 ± 1.03 | 1.65 ± 1.3 | 20.42 ± 1.27 | 32.7 ± 1.36 | 50 ± 4.71 | 44.9 ± 2.89 |
| *Alnus* | 16.49 ± 1.52 | 17.24 ± 3.4 | 2762± 2.99 | 0.31 ± 1.27 | 496.82 ± 1.07 | 1.83 ± 1.38 | 30.59 ± 1.29 | 2.41 ± 1.72 | 1563 ± 4.32 | 1.12 ± 5.04 |
| Apiaceae | 21.92 ± 1.53 | 0.59 ± 2.19 | 2445± 4.43 | 0.2 ± 1.3 | 438.76 ± 1.05 | 2.78 ± 1.45 | 25.81 ± 1.29 | 5.04 ± 1.66 | 1466 ± 9.97 | 2.53 ± 3.25 |
| *Artemisia*-type | 20.12 ± 1.62 | 0.76 ± 3.48 | 294± 4.5 | 0.24 ± 1.34 | 437.98 ± 1.05 | 2.3 ± 1.41 | 25.12 ± 1.41 | 1.25 ± 1.77 | 18184 ± 9.26 | 0.15 ± 7.71 |
| Asteraceae | 20.59 ± 1.42 | 0.58 ± 2.17 | 1236± 3.8 | 0.18 ± 1.4 | 434.69 ± 1.06 | 2.24 ± 1.43 | 22.08 ± 1.31 | 3.87 ± 2.08 | 1334 ± 6.17 | 0.63 ± 5.95 |
| *Betula* | 13.82 ± 1.31 | 8.96 ± 5.48 | 630± 4.76 | 0.38 ± 1.16 | 525.57 ± 1.05 | 1.94 ± 1.34 | 24.31 ± 1.23 | 3.01 ± 1.43 | 1646 ± 3.24 | 0.23 ± 2.87 |
| Brassicaceae | 24.03 ± 1.4 | 0.37 ± 2.21 | 798± 5.55 | 0.13 ± 1.4 | 396.16 ± 1.06 | 2.91 ± 1.28 | 31.31 ± 1.26 | 1.61 ± 1.79 | 1110 ± 9.05 | 0.69 ± 6.02 |
| Campanulaceae | 31.03 ± 1.55 | 0.43 ± 2.43 | 334± 5.39 | 0.18 ± 1.38 | 461.01 ± 1.04 | 1.77 ± 1.29 | 27.3 ± 1.26 | 1.02 ± 1.62 | 2402 ± 6.92 | 0.09 ± 3.58 |
| *Carpinus* | 22.54 ± 1.68 | 12.74 ± 4.69 | 1767± 3.95 | 0.25 ± 1.34 | 463.13 ± 1.06 | 1.8 ± 1.65 | 19.95 ± 1.37 | 19.97 ± 2.01 | 6300 ± 6.6 | 22.75 ± 7.98 |
| Caryophyllaceae | 22.21 ± 1.47 | 0.22 ± 2.37 | 83± 6.85 | 0.17 ± 1.31 | 444.86 ± 1.04 | 1.81 ± 1.73 | 23.4 ± 1.24 | 1.1 ± 1.8 | 483 ± 6.95 | 0.26 ± 5.09 |
| *Castanea* | 13.35 ± 1.66 | 26.46 ± 4.48 | 8338± 4.09 | 0.34 ± 1.36 | 495.61 ± 1.06 | 1.83 ± 1.53 | 22.18 ± 1.39 | 4.55 ± 2.02 | 1 ± 6.49 | 5583.06 ± 7.74 |
| Cerealia | 23.3 ± 1.49 | 1.16 ± 3.32 | 2589± 6.82 | 0.23 ± 1.35 | 403.39 ± 1.08 | 1.85 ± 1.84 | 24.91 ± 1.36 | 13.57 ± 2.16 | 157 ± 5.16 | 26.51 ± 7.24 |

Table 3 (continued).

| **Pollen taxon** | **SLA** | **Plant height** | **LA** | **LDMC** | **Leaf carbon** | **Leaf phosphorus** | **Leaf nitrogen** | **Seed length** | **Seed count** | **Seed mass** |
| --- | --- | --- | --- | --- | --- | --- | --- | --- | --- | --- |
|  | mg/mm^2^ | m | mm^2^ | g/g | mg/g | mg/g | mg/g | mm |  | mg |
| Convolvulaceae | 26.26 ± 1.57 | 0.59 ± 4.35 | 1455± 3.89 | 0.17 ± 1.29 | 446.34 ± 1.05 | 1.92 ± 1.42 | 30.02 ± 1.35 | 2.68 ± 2.16 | 141 ± 5.83 | 5.86 ± 9.3 |
| Cornaceae | 20.1 ± 1.53 | 2.78 ± 4.39 | 1990± 3.22 | 0.32 ± 1.28 | 444.38 ± 1.05 | 1.86 ± 1.5 | 18.58 ± 1.28 | 8.26 ± 1.77 | 13 ± 4.55 | 54.77 ± 5.42 |
| *Corylus* | 19.07 ± 1.65 | 4.45 ± 3.5 | 3733± 3.12 | 0.38 ± 1.28 | 448.41 ± 1.05 | 2.16 ± 1.35 | 20.9 ± 1.3 | 16.54 ± 1.76 | 1439 ± 4.62 | 786.07 ± 5.27 |
| Cyperaceae | 16.36 ± 1.49 | 0.34 ± 2.03 | 427± 3.37 | 0.32 ± 1.22 | 453.15 ± 1.05 | 1.35 ± 1.47 | 18.04 ± 1.26 | 2.73 ± 1.62 | 128 ± 6.71 | 0.71 ± 2.77 |
| Ericales | 10.89 ± 1.55 | 0.48 ± 3.89 | 63± 12.46 | 0.35 ± 1.24 | 542.2 ± 1.05 | 1.02 ± 1.46 | 13.75 ± 1.3 | 1.83 ± 2.35 | 378 ± 11.18 | 0.11 ± 8.64 |
| Fabaceae | 21.31 ± 1.44 | 0.54 ± 3.29 | 211± 4.49 | 0.23 ± 1.28 | 473.81 ± 1.04 | 1.65 ± 1.33 | 36.05 ± 1.23 | 2.88 ± 1.86 | 121 ± 5.76 | 6 ± 6.43 |
| *Fagus* | 14.44 ± 1.47 | 29.57 ± 2.35 | 2352± 2.27 | 0.33 ± 1.25 | 493.39 ± 1.04 | 1.29 ± 1.32 | 23.33 ± 1.24 | 7.39 ± 1.5 | 246 ± 3.25 | 226.81 ± 3.25 |
| *Fraxinus* | 14.18 ± 1.58 | 16.29 ± 3.17 | 2612± 4.15 | 0.34 ± 1.22 | 446.28 ± 1.05 | 2.02 ± 1.32 | 20.48 ± 1.27 | 31.07 ± 1.57 | 580 ± 3.68 | 52.57 ± 3.81 |
| *Humulus* | 24.39 ± 3.38 | 4.29 ± 39.02 | 5602± 29.98 | 0.29 ± 2.08 | 475.2 ± 1.16 | 1.02 ± 2.31 | 18.05 ± 2.04 | 2.51 ± 5.62 | 38 ± 93.6 | 3.36 ± 143.93 |
| *Juglans* | 15.16 ± 1.76 | 28.21 ± 5.26 | 3718± 4.78 | 0.3 ± 1.4 | 510.95 ± 1.07 | 2.38 ± 1.58 | 22.24 ± 1.43 | 3.5 ± 2.22 | 143 ± 7.85 | 9915.4 ± 9.61 |
| *Juniperus*-type | 4.55 ± 2.12 | 5.78 ± 4.5 | 21± 3.63 | 0.47 ± 1.3 | 512.12 ± 1.08 | 1.02 ± 1.52 | 11.05 ± 1.39 | 5.09 ± 1.86 | 240 ± 5.39 | 21.6 ± 6.8 |
| Lamiaceae | 23.8 ± 1.59 | 0.37 ± 2.13 | 423± 4.53 | 0.2 ± 1.34 | 449.43 ± 1.05 | 2.15 ± 1.61 | 21.32 ± 1.38 | 1.6 ± 1.57 | 303 ± 5.72 | 0.7 ± 3.61 |
| *Larix* | 9.78 ± 1.49 | 39.33 ± 3.2 | 18± 3.12 | 0.36 ± 1.27 | 511.08 ± 1.05 | 1.83 ± 1.43 | 18.51 ± 1.33 | 8.44 ± 1.73 | 131645 ± 4.41 | 4.53 ± 5.11 |
| Liliaceae-type | 21.58 ± 1.7 | 0.38 ± 5.44 | 776± 5.45 | 0.12 ± 1.46 | 439.25 ± 1.07 | 2.24 ± 1.5 | 32.83 ± 1.51 | 4.66 ± 2.4 | 127 ± 10.52 | 2.7 ± 9.62 |

Table 3 (continued).

| **Pollen taxon** | **SLA** | **Plant height** | **LA** | **LDMC** | **Leaf carbon** | **Leaf phosphorus** | **Leaf nitrogen** | **Seed length** | **Seed count** | **Seed mass** |
| --- | --- | --- | --- | --- | --- | --- | --- | --- | --- | --- |
|  | mg/mm^2^ | m | mm^2^ | g/g | mg/g | mg/g | mg/g | mm |  | mg |
| Moraceae | 18.62 ± 2.07 | 10.96 ± 5.34 | 7758± 4.67 | 0.3 ± 1.39 | 439.68 ± 1.07 | 1.75 ± 1.7 | 25.22 ± 1.43 | 2.23 ± 2.09 | 977 ± 8.53 | 4.07 ± 11.99 |
| Orobanchaceae | 20.75 ± 1.38 | 0.24 ± 2.52 | 147± 3.13 | 0.2 ± 1.27 | 452.99 ± 1.06 | 2.32 ± 1.55 | 29.63 ± 1.45 | 1.84 ± 2.45 | 276 ± 8.95 | 0.47 ± 11.2 |
| *Picea* | 4.03 ± 1.28 | 39.28 ± 1.67 | 33± 1.69 | 0.42 ± 1.15 | 494.74 ± 1.02 | 1.36 ± 1.34 | 11.41 ± 1.25 | 8.03 ± 1.31 | 115524 ± 1.91 | 4.99 ± 2.15 |
| *Pinus* | 4.55 ± 1.24 | 27.56 ± 1.51 | 67± 1.61 | 0.37 ± 1.09 | 510.08 ± 1.03 | 1.09 ± 1.33 | 11.72 ± 1.19 | 7.93 ± 1.2 | 1092 ± 1.73 | 13.97 ± 2.68 |
| Plantaginaceae | 22.57 ± 1.45 | 0.25 ± 2.19 | 366± 5.27 | 0.18 ± 1.35 | 455.57 ± 1.08 | 2.09 ± 1.47 | 20.99 ± 1.28 | 1.35 ± 1.54 | 829 ± 7.51 | 0.24 ± 3.58 |
| Poaceae | 20.45 ± 1.47 | 0.47 ± 2.03 | 454± 3.51 | 0.31 ± 1.28 | 444.99 ± 1.04 | 1.56 ± 1.61 | 19.54 ± 1.33 | 4.11 ± 1.89 | 847 ± 7.73 | 0.54 ± 4.18 |
| *Populus* | 13.49 ± 1.43 | 22.7 ± 2.86 | 2654± 2.85 | 0.38 ± 1.26 | 495.68 ± 1.06 | 1.76 ± 1.43 | 22.18 ± 1.34 | 1.68 ± 1.64 | 1588 ± 3.65 | 0.25 ± 5.18 |
| *Quercus* | 10.12 ± 1.53 | 24.42 ± 1.76 | 1973± 2.34 | 0.36 ± 1.15 | 483.86 ± 1.04 | 1.21 ± 1.37 | 18.12 ± 1.31 | 19 ± 1.27 | 176 ± 2.64 | 1903.49 ± 2.19 |
| Ranunculaceae | 22.5 ± 1.42 | 0.4 ± 3.12 | 1730± 4.01 | 0.18 ± 1.32 | 453.25 ± 1.04 | 2.32 ± 1.49 | 24.41 ± 1.24 | 3.24 ± 1.65 | 196 ± 6.35 | 1.9 ± 3.76 |
| Rosaceae | 15.96 ± 1.45 | 1.1 ± 5.26 | 1063± 3.46 | 0.34 ± 1.2 | 467.73 ± 1.04 | 1.91 ± 1.35 | 21.27 ± 1.22 | 4.99 ± 2.35 | 71 ± 16.74 | 5.18 ± 13.4 |
| Rubiaceae | 29.38 ± 1.5 | 0.35 ± 2.31 | 61± 3.79 | 0.22 ± 1.33 | 441.17 ± 1.05 | 1.89 ± 1.39 | 22.05 ± 1.29 | 1.67 ± 1.6 | 201 ± 13.3 | 1.24 ± 5.01 |
| *Rumex* | 24.61 ± 1.38 | 0.56 ± 2.63 | 1858± 4.98 | 0.13 ± 1.23 | 460.14 ± 1.04 | 2.87 ± 1.29 | 34.05 ± 1.21 | 2.27 ± 1.56 | 2070 ± 6.78 | 0.85 ± 3.3 |
| *Salix* | 12.14 ± 1.35 | 1.33 ± 7.44 | 418± 3.27 | 0.35 ± 1.2 | 491.27 ± 1.05 | 1.55 ± 1.48 | 23.53 ± 1.24 | 1.81 ± 1.7 | 8176 ± 40.58 | 0.09 ± 3.58 |
| *Sambucus nigra* | 21.34 ± 1.76 | 5.41 ± 4.85 | 920± 4.58 | 0.23 ± 1.38 | 424.91 ± 1.06 | 2.65 ± 1.46 | 37.4 ± 1.37 | 5.29 ± 2.08 | 6 ± 7 | 6.4 ± 8.68 |

Table 3 (continued).

| **Pollen taxon** | **SLA** | **Plant height** | **LA** | **LDMC** | **Leaf carbon** | **Leaf phosphorus** | **Leaf nitrogen** | **Seed length** | **Seed count** | **Seed mass** |
| --- | --- | --- | --- | --- | --- | --- | --- | --- | --- | --- |
|  | mg/mm^2^ | m | mm^2^ | g/g | mg/g | mg/g | mg/g | mm |  | mg |
| *Sanguisorba*-type | 20.48 ± 1.64 | 0.44 ± 4.62 | 318± 4.12 | 0.29 ± 1.35 | 444.08 ± 1.08 | 1.38 ± 1.44 | 20.9 ± 1.35 | 3.24 ± 1.99 | 611 ± 9.1 | 4 ± 7.88 |
| *Thalictrum* | 20.68 ± 1.85 | 0.39 ± 5.84 | 1802± 14.58 | 0.28 ± 1.43 | 447.39 ± 1.07 | 2.31 ± 1.58 | 22.94 ± 1.4 | 3.52 ± 2.29 | 412 ± 32.51 | 1.26 ± 9.66 |
| Thymelaceae | 18.25 ± 2.14 | 0.75 ± 7.06 | 351± 6.15 | 0.23 ± 1.47 | 449.37 ± 1.08 | 2.39 ± 1.81 | 24.4 ± 1.48 | 7.65 ± 2.46 | 9 ± 16.63 | 18.57 ± 18.01 |
| *Tilia* | 26.47 ± 1.64 | 16.27 ± 4.45 | 4266± 3.64 | 0.21 ± 1.31 | 462.98 ± 1.05 | 2.57 ± 1.45 | 25.86 ± 1.33 | 7.82 ± 1.89 | 1 ± 5.58 | 54.96 ± 6.43 |
| *Ulmus* | 14.74 ± 1.72 | 24.56 ± 3.57 | 2982± 3.57 | 0.31 ± 1.36 | 415.68 ± 1.05 | 1.73 ± 1.42 | 22.18 ± 1.33 | 22.19 ± 1.8 | 0 ± 4.73 | 8.04 ± 5.53 |
| *Urtica* | 30.43 ± 1.65 | 0.98 ± 4.15 | 1152± 3.9 | 0.21 ± 1.33 | 375.67 ± 1.06 | 4.11 ± 1.43 | 46.62 ± 1.35 | 1.7 ± 1.93 | 823 ± 8 | 0.23 ± 7.34 |

# References

1.

Wood, S.N. (2016). Just another gibbs additive modeller: interfacing JAGS and mgcv. *arXiv preprint arXiv:1602.02539*.

2.

Wood, S.N. (2017). *Generalized Additive Models: An Introduction with R*. 2 edn. Chapman and Hall/CRC.
